# Atomically dispersed Ir/α-MoC catalyst with high metal loading and thermal stability for water-promoted hydrogenation reaction

**DOI:** 10.1093/nsr/nwab026

**Published:** 2021-02-10

**Authors:** Siwei Li, Ruochen Cao, Mingquan Xu, Yuchen Deng, Lili Lin, Siyu Yao, Xuan Liang, Mi Peng, Zirui Gao, Yuzhen Ge, Jin-Xun Liu, Wei-Xue Li, Wu Zhou, Ding Ma

**Affiliations:** Beijing National Laboratory for Molecular Sciences, College of Chemistry and Molecular Engineering and College of Engineering, and BIC-ESAT, Peking University, Beijing 100871, China; Beijing National Laboratory for Molecular Sciences, College of Chemistry and Molecular Engineering and College of Engineering, and BIC-ESAT, Peking University, Beijing 100871, China; School of Physical Sciences and CAS Key Laboratory of Vacuum Physics, University of Chinese Academy of Sciences, Beijing 100049, China; Beijing National Laboratory for Molecular Sciences, College of Chemistry and Molecular Engineering and College of Engineering, and BIC-ESAT, Peking University, Beijing 100871, China; Beijing National Laboratory for Molecular Sciences, College of Chemistry and Molecular Engineering and College of Engineering, and BIC-ESAT, Peking University, Beijing 100871, China; Beijing National Laboratory for Molecular Sciences, College of Chemistry and Molecular Engineering and College of Engineering, and BIC-ESAT, Peking University, Beijing 100871, China; Beijing National Laboratory for Molecular Sciences, College of Chemistry and Molecular Engineering and College of Engineering, and BIC-ESAT, Peking University, Beijing 100871, China; Beijing National Laboratory for Molecular Sciences, College of Chemistry and Molecular Engineering and College of Engineering, and BIC-ESAT, Peking University, Beijing 100871, China; Beijing National Laboratory for Molecular Sciences, College of Chemistry and Molecular Engineering and College of Engineering, and BIC-ESAT, Peking University, Beijing 100871, China; Beijing National Laboratory for Molecular Sciences, College of Chemistry and Molecular Engineering and College of Engineering, and BIC-ESAT, Peking University, Beijing 100871, China; School of Chemistry and Materials Science, CAS Excellence Center for Nanoscience, Hefei National Laboratory for Physical Sciences at the Microscale, iChEM, University of Science and Technology of China, Hefei 230026, China; School of Chemistry and Materials Science, CAS Excellence Center for Nanoscience, Hefei National Laboratory for Physical Sciences at the Microscale, iChEM, University of Science and Technology of China, Hefei 230026, China; School of Physical Sciences and CAS Key Laboratory of Vacuum Physics, University of Chinese Academy of Sciences, Beijing 100049, China; CAS Center for Excellence in Topological Quantum Computation, University of Chinese Academy of Sciences, Beijing 100049, China; Beijing National Laboratory for Molecular Sciences, College of Chemistry and Molecular Engineering and College of Engineering, and BIC-ESAT, Peking University, Beijing 100871, China

**Keywords:** atomically dispersed catalysts, high metal loading, molybdenum carbide, hydrogenation of quinoline

## Abstract

Synthesis of atomically dispersed catalysts with high metal loading and thermal stability is challenging but particularly valuable for industrial application in heterogeneous catalysis. Here, we report a facile synthesis of a thermally stable atomically dispersed Ir/α-MoC catalyst with metal loading as high as 4 wt%, an unusually high value for carbide supported metal catalysts. The strong interaction between Ir and the α-MoC substrate enables high dispersion of Ir on the α-MoC surface, and modulates the electronic structure of the supported Ir species. Using quinoline hydrogenation as a model reaction, we demonstrate that this atomically dispersed Ir/α-MoC catalyst exhibits remarkable reactivity, selectivity and stability, for which the presence of high-density isolated Ir atoms is the key to achieving high metal-normalized activity and mass-specific activity. We also show that the water-promoted quinoline hydrogenation mechanism is preferred over the Ir/α-MoC, and contributes to high selectivity towards 1,2,3,4-tetrahydroquinoline. The present work demonstrates a new strategy in constructing a high-loading atomically dispersed catalyst for the hydrogenation reaction.

## Teaser

Atomically dispersed Ir/α-MoC catalyst with high metal loading exhibits remarkable activity, selectivity and stability for quinoline hydrogenation reaction through water-promoted hydrogenation mechanism.

## INTRODUCTION

Atomically dispersed catalysts have received extensive research attention [1–8] because they exhibit excellent activity and unique selectivity for many important catalytic reactions, such as CO oxidation [9–11], water gas shift reaction [12–14] and hydrogenation of organic compounds [15–20]. The atomically dispersed nature of these metal catalysts confers their unique electronic structures as well as designated coordination-unsaturated environments for optimized adsorption/activation of reactants. One grand challenge faced by these atomically dispersed catalysts is that the supported metal single-atoms are usually thermally unstable and tend to aggregate into large clusters/particles at evaluated reaction temperatures [21]. Furthermore, most reported atomically dispersed catalysts have an extremely low metal loading, below 1.5 wt% [22]. Because of the extremely low metal loading, many atomically dispersed catalysts suffer from low mass-specific activity. However, high mass-specific activity is crucial for industrial application of catalyst. Therefore, developing new strategies for constructing atomically dispersed catalysts with high metal loading, high thermal stability and high catalytic performance is of great importance.

In order to achieve high metal loading and high thermal stability, the support material should have a high specific surface area with abundant surface sites that could provide strong anchoring to the supported metal species. Meanwhile, for optimizing the catalytic performance, the support material should also be carefully chosen to tune the electronic properties of the supported species, and to participate in catalyzing the reaction [23,24]. For example, in atomically dispersed Pt/α-MoC catalyst, the α-MoC support not only endows the accommodated Pt species with the atomically dispersed nature, but also facilitates the splitting of water and the generation of hydrogen and reactive surface hydroxyl groups, which is critical for the aqueous reforming of methanol into hydrogen and CO_2_. Constructing atomically dispersed catalysts using active substrates, such as transition metal carbides and nitrides, is, therefore, crucial in catalyst design [25–28].

Herein, we report the synthesis of an atomically dispersed Ir catalyst on active α-MoC support with 4 wt% Ir loading through a facile wet-impregnation method. Additionally, the high-loading atomically dispersed Ir/α-MoC catalyst can efficiently catalyze the hydrogenation reaction of quinoline towards 1,2,3,4-tetrahydroquinoline (py-THQ), an important building block in pharmaceuticals and fine chemicals [29–46]. We observed that the α-MoC host endorsed the catalyst’s unexpected capability to block the unselective hydrogenation of the benzene ring of quinoline without sacrificing the catalytic activity under our reaction condition. The origin of the high reactivity and especially the high selectivity of the current catalyst construct, as compared with pure Ir metal catalysts, is further explained by the first-principles microkinetic simulations.

## RESULTS AND DISCUSSION

### Structure analysis of Ir/α-MoC

**Figure 1.**
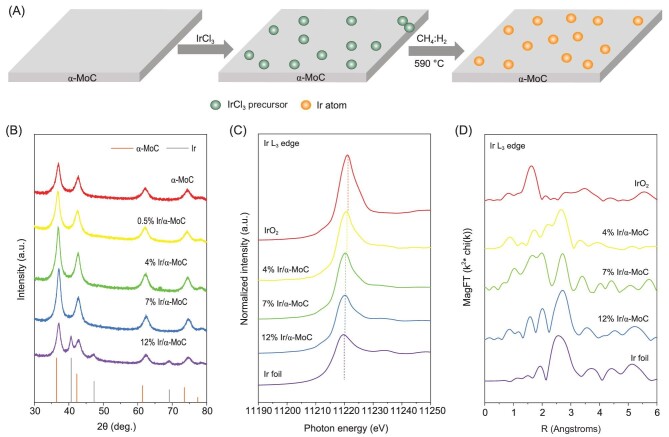
(A) Schematic illustration for the synthesis of atomically dispersed Ir/α-MoC catalyst. (B) XRD patterns of α-MoC and Ir/α-MoC catalysts with different Ir content. (C) Ir L_3_-edge X-ray adsorption near-edge spectroscopy (XANES) of the Ir/α-MoC catalysts. (D) EXAFS spectra of the Ir/α-MoC catalysts and references.

x% Ir/α-MoC (x, ranging from 0.5 to 12, is the approximate Ir loading in wt%) samples were synthesized through a facile wet-impregnation method under Ar protection, followed by carburization in CH_4_/H_2_ flow at 590^o^C (Fig. 1A). X-ray diffraction (XRD) patterns of the 0.5% Ir/α-MoC, 4% Ir/α-MoC and 7% Ir/α-MoC catalysts (Fig. 1B) match well with the standard pattern of α-MoC [23]. The absence of diffractions associated with Ir crystal indicates that the size of Ir species in these samples is below the detection limit of XRD. However, when we increase the Ir loading to 12%, besides the peaks assigned to α-MoC, new peaks associated with metallic Ir crystal appear [47], which implies that Ir aggregates into larger nanoparticles (NPs) in the 12% Ir/α-MoC catalyst.

The structure of α-MoC supported Ir species was further investigated using X-ray absorption fine structure (XAFS) characterizations. The Ir L_3_-edge XAFS (Fig. 1C) shows that the near-edge absorption energy of Ir on α-MoC is located between those of Ir foil and IrO_2_, suggesting that the Ir species is partially positively charged in these Ir/α-MoC catalysts, which demonstrates the strong interaction between α-MoC and supported Ir species. The extended X-ray absorption fine structure (EXAFS, Fig. 1D) spectra and the corresponding fitting results (Fig. S1 and Table S1) show the absence of Ir-Ir scattering in the 4% Ir/α-MoC, indicating full atomic dispersion of Ir over α-MoC. The coordination number of Ir-Mo and Ir-C shell is 7.3 and 1.4 for 4% Ir/α-MoC, respectively. The high Ir-Mo and Ir-C coordination numbers imply that Ir atoms are embedded in the Mo-terminated α-MoC surface, enabling excellent thermal stability. The coordination number of Ir-Ir (CN_Ir-Ir_) increases to 2.6 for 7% Ir/α-MoC, indicating that small Ir clusters start to form at this Ir loading. For 12% Ir/α-MoC, CN_Ir-Ir_ further increases to 4.2 with the formation of XRD-detectable Ir NPs.

**Figure 2.**
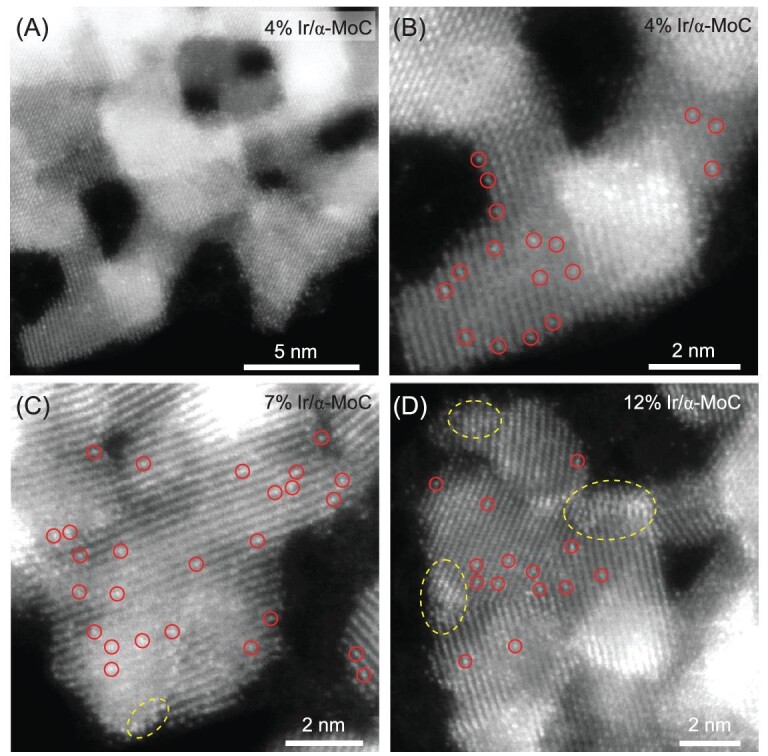
(A) A typical STEM-HAADF image of the 4% Ir/α-MoC sample. (B–D) Atomic-resolution STEM-HAADF images of Ir/α-MoC samples with different Ir loading. Atomically dispersed Ir atoms are marked with circles while Ir clusters are indicated by dashed yellow ellipses.

To further unravel the structure of Ir/α-MoC catalysts, aberration corrected scanning transmission electron microscopy (STEM) analysis was performed. As shown by the typical STEM image of 4% Ir/α-MoC sample (Fig. 2A), the α-MoC support, with an average grain size of ∼5 nm, preserves as a porous matrix even after Ir loading. Some isolated atomic sites with high contrast (highlighted in red circles, marked as Ir_1_) can be clearly distinguished in the STEM annular dark field (ADF) images (Fig. 2B), which can be identified as Ir_1_ atoms supported on α-MoC (Fig. S2). When the Ir loading is increased to 7%, the surface density of the Ir_1_ atoms also increases considerably (Fig. 2C), and no severe Ir segregation was observed in the corresponding energy-dispersive X-ray spectroscopy (EDS) mapping (Fig. S3B). The high-resolution electron energy loss spectroscopy (EELS) mapping result (Fig. S3E), however, reveals the formation of very small Ir clusters (masked with the dashed yellow circles in Figs 2C and S3D) in the 7% Ir/α-MoC sample, which is consistent with the EXAFS fitting results. Notably, despite the emergence of Ir clusters, the 7% Ir/α-MoC still has a higher Ir_1_ density than that of the 4% Ir/α-MoC (Fig. S4). Even for the 12% Ir/α-MoC sample, a considerable density of isolated Ir_1_ atoms are still observed (Fig. 2D), with the coexistence of Ir clusters and NPs as revealed by STEM-EDS and STEM-EELS mappings (Figs S5 and S6). Density functional theory (DFT) calculations reveal that the formation of Ir_1_/α-MoC is slightly endothermic by 0.36 eV with respect to the defective α-MoC (111) surface and an Ir atom in bulk, demonstrating the high possibility of the formation of an atomically dispersed Ir/α-MoC catalyst. The DFT results are in excellent agreement with our experimental observation that an ultra-high density of atomically dispersed Ir dominates on α-MoC support even at a high Ir loading of 7%.

### Catalytic performance of Ir/α-MoC towards quinoline hydrogenation

We evaluated Ir/α-MoC catalysts for the hydrogenation reaction of quinoline towards py-THQ at 120^o^C and 3 MPa (reaction time: 1 hour). Over-hydrogenation of py-THQ will produce low value decahydroquinoline (DHQ), especially under relatively high temperature and long reaction time. As seen in Table 1, the bare α-MoC exhibits poor activity (entry 1), whereas the activity for quinoline hydrogenation is significantly enhanced after the introduction of 0.5% of Ir (entry 2, 16% conversion). Conversion of quinoline dramatically increases to 85% when the Ir loading is increased to 4% (entry 3), which strongly suggests that the activation of quinoline occurs over the Ir motif. Significantly, the selectivity is unchanged, i.e. there is no formation of DHQ even at this high conversion of quinoline. When the Ir loading is further increased to 7%, a near unity conversion is reached but again no DHQ is detected (entry 4). To our surprise, when the Ir loading is 12%, while the selectivity remains unchanged, the conversion drops to 77% (entry 5).

**Table 1.** The catalytic performance of Ir/α-MoC and Ir/C in the hydrogenation reaction of quinoline.^a^ 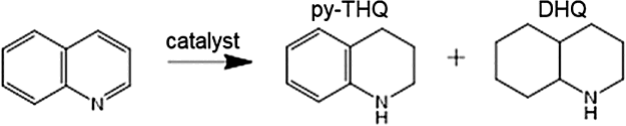

Entry	Catalyst	T (^o^C)	t (hrs)	Conv. (%)	Select. of py-THQ (%)	Select. of DHQ (%)
1	α-MoC	120	1	3	>99	n.d.^b^
2	0.5% Ir/α-MoC	120	1	16	>99	n.d.
3	4% Ir/α-MoC	120	1	81	>99	n.d.
4	7% Ir/α-MoC	120	1	95	>99	n.d.
5	12% Ir/α-MoC	120	1	77	>99	n.d.
6	7% Ir/C	120	1	97	94	6

^a^Reaction condition: catalyst (30 mg), 40 mg quinoline, 3 mL of CH_3_OH/H_2_O (v: v = 1 : 1) as solvent, 3.0 MPa of H_2_. ^b^Not detected (n.d.) by gas chromatography-mass spectrometer (GC-MS).

**Figure 3.**
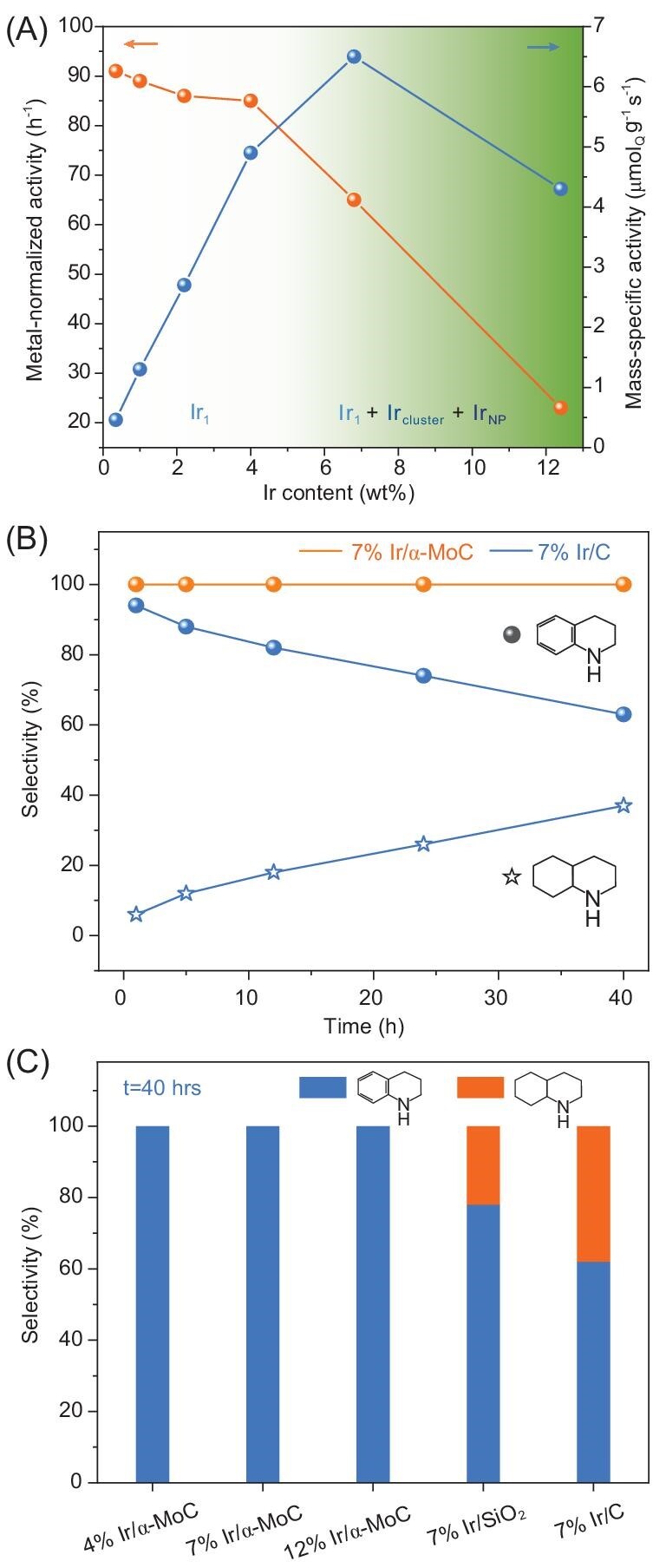
(A) Metal-normalized activity and mass-specific activity of Ir/α-MoC catalysts with different Ir content. Ir_1_ stands for atomically dispersed Ir. The shading highlights that when Ir loading is lower than 4%, the dominant species is Ir_1_; it gradually changes the mixture of Ir_1_, Ir clusters and Ir nanoparticles at higher Ir loading. (B) Time-dependent selectivity for hydrogenation of quinoline over 7% Ir/α-MoC and 7% Ir/C catalysts. (C) The selectivity of quinoline hydrogenation over Ir/α-MoC and reference Ir-based catalysts at 40 h.

To further understand the catalytic performance of the Ir/α-MoC catalysts with different loading of Ir, we plot the metal-normalized activity and mass-specific activity of a series of Ir/α-MoC catalysts at 20% conversion of quinoline as a function of Ir loading (Fig. 3A, Table S2). While the metal-normalized activity indicates the intrinsic activity of the catalytically active sites, the mass-specific activity represents the total power to convert the reactant over a catalyst with given mass, which is sometimes more important for industrial application. When the Ir content is lower than 4%, where atomically dispersed Ir_1_ dominates on the α-MoC surface, a constant metal-normalized activity (85–91 h^–1^) is observed, suggesting that the atomically dispersed Ir_1_ species has a similar intrinsic activity. As shown in Fig. 3A (right column), the mass-specific activity increases almost linearly with Ir content from 0.46 μmol_Q_ g^–1^ s^–1^ (0.5% Ir/α-MoC; Q refers to quinoline) to 4.9 μmol_Q_ g^–1^ s^–1^ (4% Ir/α-MoC). This further demonstrates that the atomically dispersed Ir_1_ species has almost the same intrinsic reactivity, and increasing the density of Ir_1_ species leads to a linear increase in mass-specific activity.

Further increasing the loading of Ir, a different scenario was observed. When the loading of Ir increased to 7%, the metal-normalized activity decreased to 65 h^–1^. As shown in Figs 1 and 2, for the 7% Ir/α-MoC catalyst, Ir clusters begin to appear, alongside with the Ir_1_ species. The accompanied decrease in metal-normalized activity with the emergence of Ir clusters indicates that Ir_1_ is more effective in the reaction than the aggregated Ir species. However, the mass-specific activity of 7% Ir/α-MoC (6.5 μmol_Q_ g^–1^ s^–1^) is still higher than that of 4% Ir/α-MoC due to the higher Ir_1_ density of the 7% Ir/α-MoC catalyst (Fig. S4), which again signals the importance of the presence of high density Ir_1_ species for the reaction. When the loading of Ir increases to 12%, a sharp decrease of metal-normalized activity (23 h^–1^) is found, which is attributed to the aggregation of Ir_1_ to Ir NPs, leading to the decrease in Ir_1_ density. As a result, the mass-specific activity decreases to 4.3 μmol_Q_ g^–1^ s^–1^. This explains also the drop in conversion in Table 1 when the Ir loading reaches 12%. This observation demonstrates that increasing the loading of noble metal cannot always ensure a higher total power for the conversion of the reactants while an optimized dispersion/surface conformation is critical for the efficient utilization of noble metal resources.

Based on the results above, we can draw the conclusion that the Ir_1_ species on α-MoC surface is more reactive than Ir clusters or Ir NPs in this reaction, giving the highest metal-normalized activity on 0.5–4% Ir/α-MoC catalysts. We need to point out that very low metal loading of a supported metal catalyst can result in an extremely low mass-specific activity, which is a drawback in practical applications. In our view, high-loading atomically dispersed catalysts (e.g. 4% Ir/α-MoC) and catalysts with the highest density of isolated metal atom (e.g. 7% Ir/α-MoC) are significant for both academia and chemical industry.

The selectivity towards py-THQ is another critical standard for quinoline hydrogenation catalysts, so we studied the time-dependent reaction behavior of the 7% Ir/α-MoC catalyst (Fig. 3B). Significantly, the 7% Ir/α-MoC catalyst achieves the yield of py-THQ at 94% with >99% selectivity in 1 h. Prolonging the reaction time to 40 h, the selectivity remains unchanged, which suggests that the hydrogenation of the benzene ring in quinoline is effectively blocked. For comparison, an Ir catalyst supported over active carbon (7% Ir/C catalyst) shows a high selectivity of 94% towards py-THQ at 1 h (entry 6 in Table 1), but the selectivity towards the undesired DHQ gradually increases with reaction time and reaches 37% after 40 h. The different selectivity could be due to either the difference in Ir dispersion or the presence of the α-MoC support. Differently to 7% Ir/α-MoC, 7% Ir/C catalyst is dominated with 2–3 nm Ir NPs (see Fig. S7). However, as shown in Fig. 3C, formation of undesired DHQ was not detected in the 40 h hydrogenation reaction for the 12% Ir/α-MoC catalyst with the presence of Ir NPs. This strongly indicates that the α-MoC substrate plays the dominant role for switching off the hydrogenation of the benzene ring of quinoline, which cannot be realized by using conventional support like carbon black and SiO_2_ (Fig. 3C). Moreover, 7% Ir/α-MoC shows remarkable stability with both conversion and selectivity maintained after five cycles (Fig. S8). The catalytic activity and stability of the 7% Ir/α-MoC catalyst is comparable to the traditional supported nanoparticles (Table S3) [29–32,48–50]. As tested by inductively coupled plasma-atomic emission spectrometry (ICP-AES), no Ir species was detected in the filter liquor, and Ir content in the 7% Ir/α-MoC catalyst did not change after five cycles. XRD and STEM analyses (Figs S9 and S10) confirm the good structural stability of Ir_1_ under reaction conditions.

The 7% Ir/α-MoC catalyst was further employed to catalyze the hydrogenation of quinoline derivatives with different functional groups. Only the hydrogenation of the heteroarene ring was observed in the hydrogenation of quinoline derivatives (Table S4), while the hydrogenation of the benzene ring of the quinoline derivatives was switched off effectively. Quinoline compounds bearing a methyl group at the 2- and 6- position could be completely hydrogenated to the corresponding py-THQ (Table S4, entries 2–3). Notably, the 7% Ir/α-MoC catalyst could catalyze the hydrogenation of 8-hydroxyquinoline to biologically active 1,2,3,4-tetrahydro-8-hydroxyquinoline without any dehydroxylation (Table S4, entry 4). In a more challenging reaction, 6-chloroquinoline was reduced to 6-chloro-1,2,3,4-tetrahydroquinoline with a conversion of 92% and a selectivity of 97% (Table S4, entry 5). Besides quinoline hydrogenation, the 7% Ir/α-MoC catalyst also gives an extraordinary catalytic performance for the hydrogenation of isoquinoline (Table S4, entry 6).

### First-principles microkinetic simulations on quinoline hydrogenation

**Figure 4.**
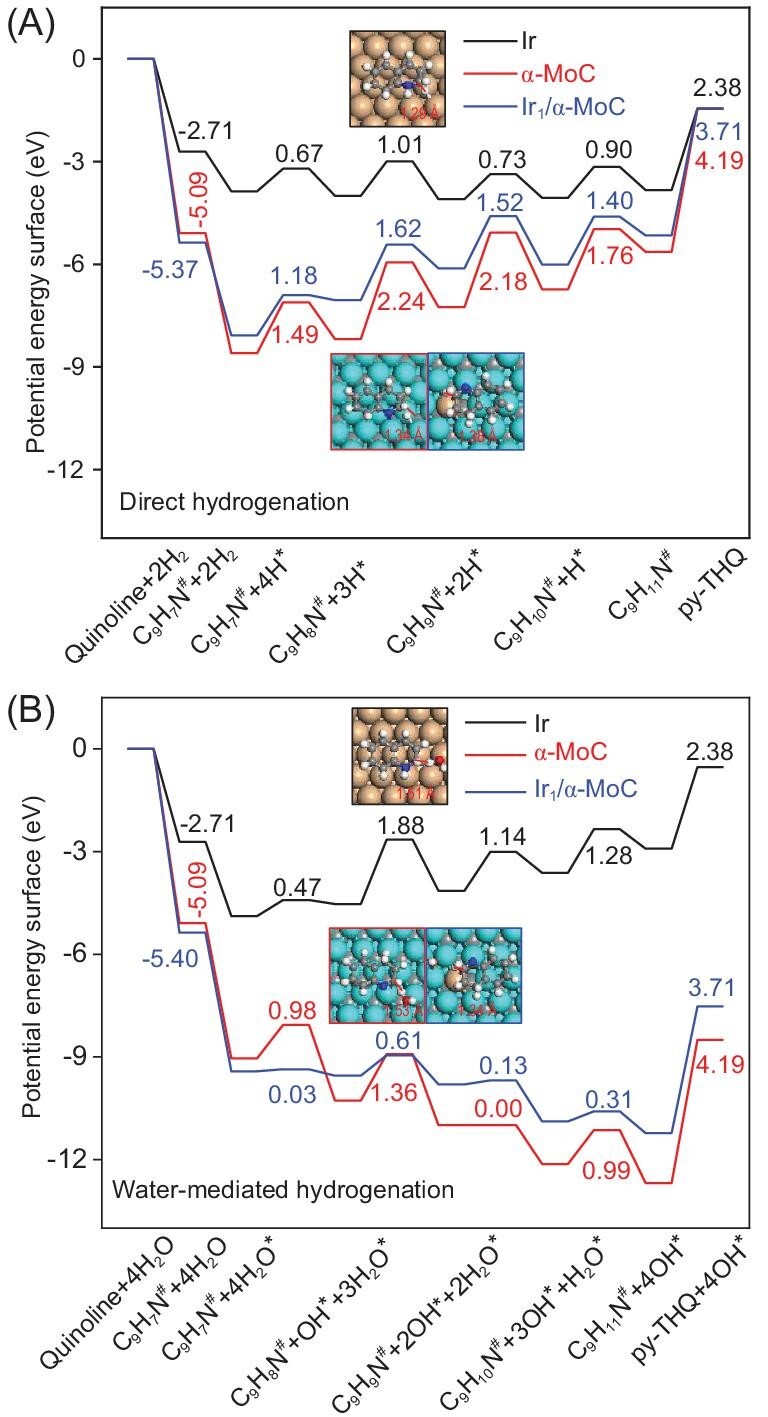
DFT calculated potential energy diagrams for (A) direct and (B) water-mediated quinoline hydrogenation towards py-THQ over Ir (111), α-MoC(111) and Ir_1_/α-MoC(111) surfaces. Quinoline/py-THQ adsorption energies and activation barriers for quinoline hydrogenation are indicated in eV. The transition state configurations for C_9_H_8_N hydrogenation are shown and the corresponding bond lengths between C and H fragments at transition states are given in Å. The cyan, orange, gray, red, blue and white spheres are Mo, Ir, C, O, N and H atoms, respectively.

To understand the excellent reactivity and selectivity of Ir/α-MoC catalysts in the hydrogenation reaction of quinoline, van der Waals (vdW) corrected DFT calculations combined with microkinetic simulations were performed to study quinoline hydrogenation mechanisms over a series of catalysts. Ir(111), α-MoC(111) and Ir_1_/α-MoC(111) models were used to represent the Ir particle catalyst, α-MoC catalyst and atomically dispersed Ir/α-MoC catalyst, respectively. In order to explore the solvent effect, a water molecule was considered in the present work to study the effect of water-mediated reaction route. Therefore, two quinoline hydrogenation mechanisms, namely direct and water-mediated quinoline hydrogenation pathways, were investigated. The calculated potential energy diagrams for quinoline hydrogenation towards py-THQ are presented in Fig. 4. As compared with Ir, quinoline and py-THQ adsorb stronger on α-MoC by 2.38 eV and 1.81 eV, respectively (Table S5), which can be attributed to a higher degree of charge transfer from metallic-like α-MoC to quinoline/py-THQ enhancing the electrostatic interaction between α-MoC and quinoline/py-THQ (Table S8). In analogy to quinoline/py-THQ adsorption, α-MoC also binds the alternative reactants, namely the H atom and H_2_O molecule, more strongly than Ir by at least 0.45 eV (Tables S5–S7). The presence of Ir_1_ atoms on the α-MoC surface further enhances quinoline adsorption while weakening py-THQ and hydrogen adsorption strengths slightly. The significantly different energetics for the adsorption of reactants and products over Ir, α-MoC and Ir_1_/α-MoC will result in distinct catalytic performances among them as described below.

We first focused on the direct quinoline hydrogenation mechanism by which all the intermediates are hydrogenated by the surface adsorbed hydrogen atoms (Figs. 4A and S11). All the direct quinoline hydrogenation steps are almost neutral on Ir with moderate hydrogenation barriers ranging from 0.67 eV to 1.01 eV. The C_9_H_8_N hydrogenation step has the highest reaction barrier on Ir. In contrast, direct quinoline hydrogenation becomes highly endothermic over α-MoC, with significantly high activation barriers ranging from 1.49 eV to 2.24 eV. Relatively lower hydrogenation reaction barriers of 1.18 eV to 1.62 eV were found on Ir_1_/α-MoC. The higher activation barriers for direct quinoline hydrogenation on α-MoC and Ir_1_/α-MoC than on Ir metal cannot rationalize the experimental finding that Ir_1_/α-MoC displays comparable activity to Ir.

In contrast to the direct hydrogenation mechanism, hydrogenation of quinoline via water-mediate becomes endothermic step-by-step on Ir (Fig. 4B) due to the weak adsorption of OH intermediate (*E*_OH_ = −2.82 eV). On Ir, the highest activation barrier in the water-mediated hydrogenation mechanism becomes 1.88 eV, which is 0.87 eV higher than that in the direct hydrogenation mechanism. Therefore, direct hydrogenation of quinoline is more favorable than the water-mediated hydrogenation pathway over Ir. However, in contrast to Ir, quinoline hydrogenation via water-mediate is exothermic continuously on both α-MoC and Ir_1_/α-MoC structures (Fig. 4B). Consequently, the activation barriers for water-mediated hydrogenation mechanism become significantly lower than those in the direct hydrogenation route. In other words, water-mediated quinoline hydrogenation mechanism is more favorable than the direct hydrogenation route on both α-MoC and Ir_1_/α-MoC catalysts. Ir_1_/α-MoC has lower hydrogenation barriers than α-MoC because of their different transition state configurations in the water-mediated quinoline hydrogenation mechanism. On α-MoC, water molecules serve as a whole species to react with quinoline and the corresponding intermediates (Figs. 4B and S12). However, on Ir_1_/α-MoC, the detached H atom from water reacts with quinoline and C_9_H_8_N directly with the counterpart OH species moving away to Mo sites (Fig. S13), which lowers the activation barriers for quinoline hydrogenation. Importantly, the activation barriers for each water-mediated quinoline hydrogenation step over Ir_1_/α-MoC are at least 0.40 eV lower than those in the direct hydrogenation mechanism on Ir metal.

**Figure 5.**
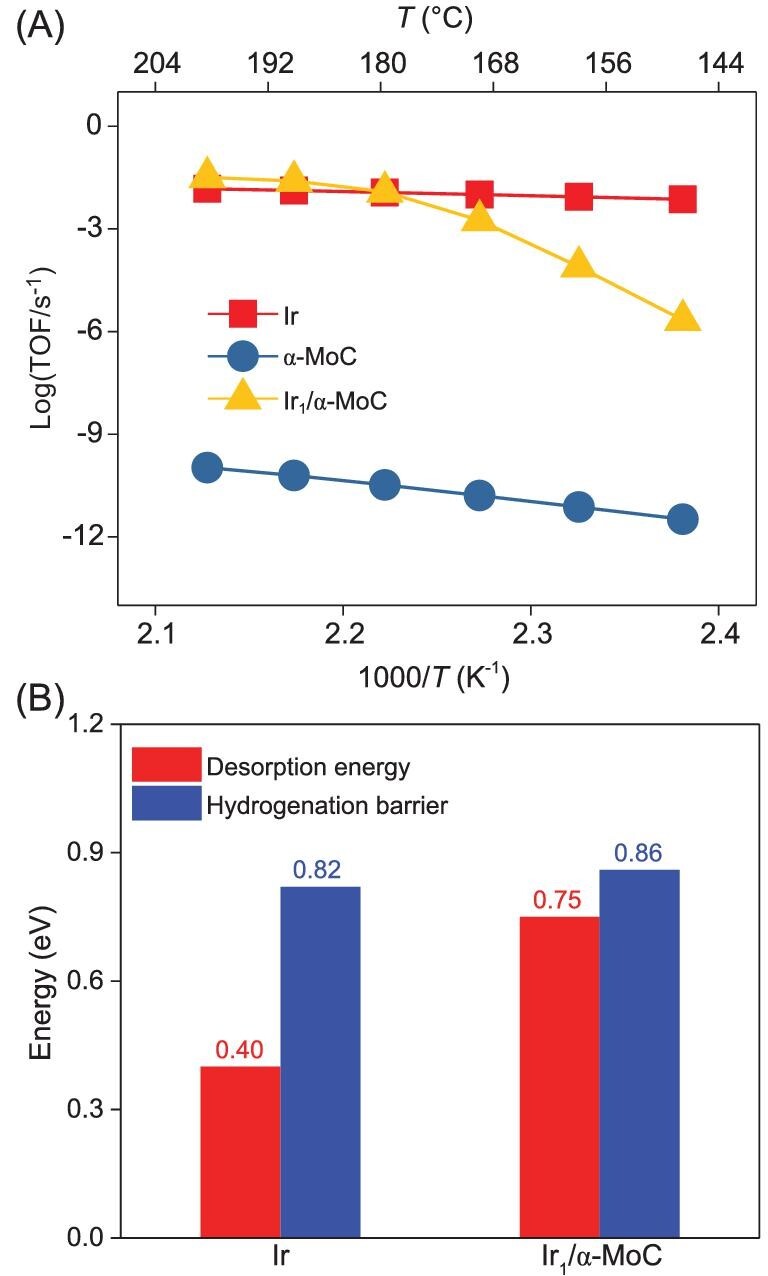
(A) Predicted reaction rate (TOF, s^–1^) of quinoline hydrogenation towards py-THQ over Ir, α-MoC and Ir_1_/α-MoC surfaces by microkinetic simulations. (B) DFT calculated activation barrier for py-THQ hydrogenation and desorption energy of py-THQ over Ir and Ir_1_/α-MoC surfaces. All energies are indicated in eV unit and py-THQ desorption energy is normalized per site.

To quantify the catalytic performance of Ir, α-MoC and Ir_1_/α-MoC catalysts, microkinetic simulations were performed. The theoretically predicted activity trend for quinoline hydrogenation, i.e. Ir ∼ Ir_1_/α-MoC > α-MoC, is corroborated by our experimental measurements (Fig. 5A). By possessing moderate hydrogenation activation barriers and surface coverage of H, the Ir catalyst has an extraordinary activity for quinoline hydrogenation and direct hydrogenation of C_9_H_8_N is the rate-determining step (RDS) (Fig. S14). α-MoC has the lowest reaction rate and the water-mediated hydrogenation of quinoline (C_9_H_7_N) is the RDS. H_2_O dissociation is highly exothermic on α-MoC. Therefore, H_2_O dissociation is feasible on α-MoC and the α-MoC surface is fully covered by OH/H species, leaving fewer active sites for the adsorption of quinoline. The high activation barrier of C_9_H_7_N hydrogenation and low surface coverage of C_9_H_7_N (Fig. S14) both result in a significantly low activity of quinoline hydrogenation of α-MoC. Additionally, quinoline hydrogenation activity is low on Ir_1_/α-MoC catalyst at relatively low temperatures. However, the reaction rate increases drastically at a higher temperature (from 120°C to 180°C), where C_9_H_8_N hydrogenation via water-mediate is identified as the RDS. Ir_1_/α-MoC has comparable quinoline hydrogenation activity to the Ir catalyst at temperatures above 180°C but with a different quinoline hydrogenation mechanism.

The water effect in quinoline hydrogenation was investigated by combining water with different kinds of neat solvents. The metal-normalized activities over the 7% Ir/α-MoC catalyst using methanol/water, ethanol/water and N,N-dimethylformamide (DMF)/water as solvent are 65, 72 and 7.7 h^–1^, respectively, all higher than that using neat methanol (33 h^–1^), ethanol (53 h^–1^) or DMF (3.6 h^–1^) solvent (Fig. S15). This strongly indicates that water promotes the hydrogenation of quinoline over Ir/α-MoC catalysts. In contrast, the addition of water into methanol does not improve the metal-normalized activity of the 7% Ir/C catalyst. These results suggest that direct quinoline hydrogenation is preferable on the Ir/C catalyst while water plays an import role in quinoline hydrogenation over the Ir/α-MoC catalyst; this is corroborated by our theoretical calculations.

**Scheme 1.**
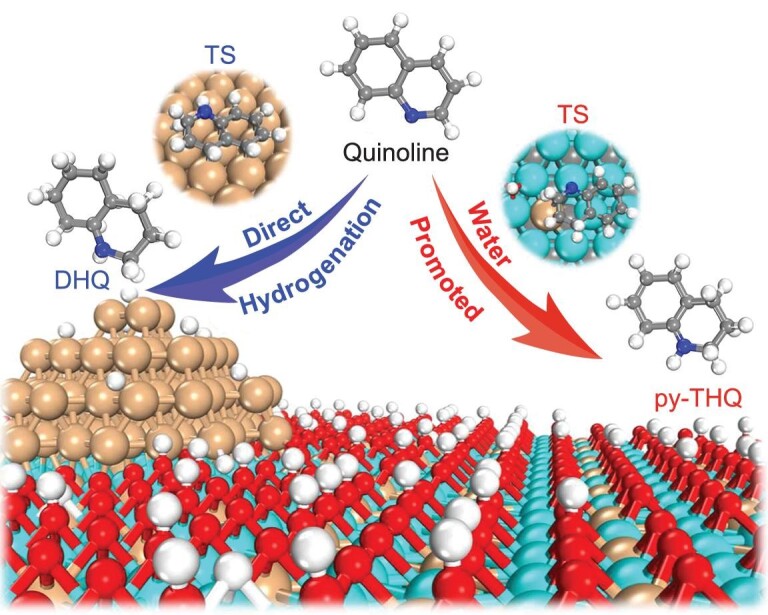
The different mechanisms for quinoline hydrogenation over Ir and Ir_1_/α-MoC surfaces. Direct hydrogenation of quinoline is preferable over Ir particles in the formation of DHQ, whereas water-promoted quinoline hydrogenation pathway is more favorable on Ir_1_/α-MoC catalyst with a high selectivity toward py-THQ. The cyan, orange, gray, red, blue and white spheres are Mo, Ir, C, O, N and H atoms, respectively. Transition state is abbreviated as TS.

The unexpected good selectivity of quinoline hydrogenation over the Ir/α-MoC catalyst is evaluated by comparing the competition between py-THQ desorption and further hydrogenation. On Ir, the calculated hydrogenation barrier and desorption energy of py-THQ are 0.82 and 0.40 eV per site, respectively (Fig. 5B). This indicates that py-THQ prefers desorption over hydrogenation, in line with our experimental observation that the selectivity of py-THQ is high at the initial stage of quinoline hydrogenation. Due to the presence of abundant empty sites on pure Ir, py-THQ could re-adsorb at the surface active sites for further hydrogenation to DHQ. Therefore, the selectivity towards DHQ will increase with prolonged reaction time over Ir catalyst. However, the remarkable selectivity of quinoline hydrogenation towards py-THQ is highlighted on Ir_1_/α-MoC even under long reaction time. On one hand, the calculated activation barrier for py-THQ further hydrogenation is 0.86 eV which is 0.11 eV higher than py-THQ desorption energy (Fig. S16). This demonstrates that the generated py-THQ product will desorb rather than undergo further hydrogenation resulting in a high selectivity of py-THQ on the Ir_1_/α-MoC catalyst. On the other hand, the surface of Ir_1_/α-MoC tends to be covered with O/OH/H species (Fig. S14) via water dissociation, leaving no sufficient empty sites for py-THQ re-adsorption and further hydrogenation. Quinoline hydrogenation can proceed quickly on Ir_1_/α-MoC due to its stronger adsorption strength and lower hydrogenation reaction barriers compared to py-THQ. In other words, the selectivity towards DHQ by quinoline hydrogenation on Ir_1_/α-MoC is significantly low due to the weak adsorption of py-THQ and lack of a sufficient empty active site for py-THQ re-adsorption for further hydrogenation (Scheme 1).

## CONCLUSION

In this work, we demonstrate the construction of an Ir/α-MoC catalyst with a high density of atomically dispersed Ir species (up to 4%) for hydrogenation reaction of quinoline to py-THQ. XAFS and STEM characterization demonstrate that the 4% Ir/α-MoC catalyst realizes full atomic dispersion of Ir, whereas the 7% Ir/α-MoC has the highest density of isolated Ir_1_ atoms on α-MoC with the co-existence of small Ir clusters. The reaction data indicates that high-density isolated Ir atoms are the key factor in acquiring remarkable metal-normalized activity and mass-specific activity, whereas the α-MoC host contributes to blocking the unselective hydrogenation of the benzene ring in quinoline at harsh reaction conditions (120^o^C, 40 h). First-principles microkinetic simulations reveal that the water-mediated hydrogenation mechanism is dominant on Ir/α-MoC catalysts as opposed to the direct hydrogenation pathway on Ir catalysts. The quinoline hydrogenation activity of Ir/α-MoC is comparable to that of Ir, due to the low hydrogenation barriers over Ir/α-MoC in the water-mediated hydrogenation pathway. For Ir/α-MoC catalysts, the high selectivity of quinoline hydrogenation towards py-THQ originates from the weak adsorption of py-THQ and the lack of sufficient empty active sites for py-THQ re-adsorption and hydrogenation. The present work not only reports a strategy to construct an atomically-dispersed Ir/α-MoC catalyst with high metal loading and thermal stability, but also provides a new strategy for improving the selectivity of a chemical reaction by selectively switching off some of the undesired reaction path using carbide supported metal catalysts.

## METHODS

α-MoC support was synthesized via a temperature progress annealing of MoO_3_. 0.8 g of MoO_3_ powder loaded in a quartz tube reactor was placed in a vertical furnace. Then, the powder was heated to 700^o^C in NH_3_ flow (160 mL/min) at a rate of 5°C/min and held at 700°C for 2 h. After cooling to room temperature (RT), NH_3_ was switched by CH_4_/H_2_ mixture (100 mL/min; 20/80 v/v). The temperature was increased to 700^o^C (5°C/min) and held for 2 h. Finally, the sample was cooled to RT and passivated with 0.5% O_2_/Ar gas.

Ir/α-MoC with different Ir content was synthesized via the wet-impregnation method under Ar atmosphere. Taking 7% Ir/α-MoC as an example, 100 mg of α-MoC powder and 1 mL of 20 mg/mL IrCl_3_ aqueous solution were mixed. After stirring for 2 h, the water was evaporated and the sample was frozen and dried overnight. Then the sample was treated in 20 mL/min of CH_4_/H_2_ mixture (15/85 v/v) at 590°C for 2 h. After cooling to RT, the sample was transferred into the reaction mixture without exposure to atmosphere for catalytic test.

## Supplementary Material

nwab026_Supplemental_File
