# Supplementary material for: Atomically dispersed Ir/α-MoC catalyst with high metal loading and thermal stability for water-promoted hydrogenation reaction
Source: Natl Sci Rev. 2021 Feb 10;9(1):nwab026. doi: 10.1093/nsr/nwab026 (PMC8794590; doi:10.1093/nsr/nwab026)
Supplement: nwab026_Supplemental_File [file nwab026_supplemental_file.docx]

**Atomically dispersed Ir/**α-**MoC catalyst with high metal loading and thermal stability for water-promoted hydrogenation reaction**

Siwei Li^a+^, Ruochen Cao^a+^, Mingquan Xu^b+^, Yuchen Deng^a^, Lili Lin^a^, Siyu Yao^a^, Xuan Liang^a^, Mi Peng^a^, Zirui Gao^a^, Yuzhen Ge^a^, Jin-Xun Liu^c*^, Wei-Xue Li^c^, Wu Zhou^b,d*^ and Ding Ma^a*^

^a^ Beijing National Laboratory for Molecular Sciences, College of Chemistry and Molecular Engineering and College of Engineering, and BIC-ESAT, Peking University, Beijing 100871, P. R. China.

^b^ School of Physical Sciences and CAS Key Laboratory of Vacuum Physics, University of Chinese Academy of Sciences, Beijing 100049, China.

^c^ School of Chemistry and Materials Science, CAS Excellence Center for Nanoscience, Hefei National Laboratory for Physical Sciences at the Microscale, iChEM, University of Science and Technology of China, Hefei 230026

^d^ CAS Center for Excellence in Topological Quantum Computation, University of Chinese Academy of Sciences, Beijing 100049, China.

^+^ These authors contribute equally to this work.

* Corresponding authors: Jin-Xun Liu (jxliu86@ustc.edu.cn), Wu Zhou ([wuzhou@ucas.ac.cn](mailto:wuzhou@ucas.ac.cn)), Ding Ma ([dma@pku.edu.cn](mailto:dma@pku.edu.cn)).

***Characterization.*** The electron-transparent STEM specimens were prepared by ultramicrotomy after embedding the powders into resin, and were baked at 100 °C in vacuum for 20 hrs before loaded into the electron microscope. STEM characterization was performed on an aberration-corrected Nion HERMES-100 under the accelerating voltage of 60 kV. The convergence angle of probe-forming aperture is set to 32 mrad. The collection angle of BF and ADF imaging is 0-10 mrad and 72-210 mrad, respectively. The EELS mappings were acquired under the same condition with a collection angle of 72 mrad. The STEM energy-dispersive X-ray spectroscopy (EDS) analysis were performed on a Titan Cubed Themis G2 300 instrument with a super-X EDS system. A probe setting with high current was used to increase the EDS signals from the small Ir clusters, which would result in a slightly worse spatial resolution due to the larger probe size. XRD analysis was performed on a PANalytical X’Pert Powder X-ray diﬀractometer, which was equipped with Cu K_α_ radiation. For XRD and STEM test, Ir/α-MoC was passivated with 0.5% O_2_/Ar gas. The accelerating current and voltage were 40 mA and 40 kV respectively. XAFS experiments were operated at the BL14W beamline at Shanghai Synchrotron Radiation Facility (SSRF). All collected spectra were analyzed using Athena and Artemis program within the Ifeffit package.

***General procedure for hydrogenation reaction.*** The hydrogenation of quinoline was investigated in a 10 mL stainless steel high-pressure batch reactor. Typically, quinoline (0.5 mmol) and the solvent (3 mL) were loaded into the autoclave and then catalyst powder was transferred into the mixture quickly without exposure to atmosphere. Firstly, the reactor was purged four times with pure H_2_ to remove air. Afterwards, the reactor was charged with 3 MPa of H_2_ and the reaction mixture was stirred with a speed of 800 r.p.m at 120 ^o^C for different reaction period. After the reaction had reached completion, the reaction products were analyzed by a GC (Agilent, 7820A) equipped with a flame ionization detector (FID) using 1,4 dioxaneas the internal standard, as well as by GC−MS (Agilent 5975C with triple-axis detector). To investigate the effect of water, neat MeOH, EtOH, DMF and their mixture with water were employed as solvents (Figure S15). To calculate metal-normalized activity (MNA) and mass-specific activity (MSA) of the samples in Figure 3, the conversion was controlled below 20% by changing the amount of quinoline, as seen in Table S2.

$\mathrm{MNA}=\frac{192*n(Quinoline )}{m\left( Ir \right)wt(Ir)t}$ (S1)

$MSA=\frac{n(Quinoline)}{m(catalyst)t}$ (S2)

***Computational Details.*** All DFT calculations were performed by using projector augmented wave (PAW)(1) potentials and the Perdew–Burke–Ernzerhof (PBE) functional(2) as implemented in the Vienna Ab initio Simulation Package (VASP).(3, 4) The effect of van der Waals (vdW) interaction was taken into account by using the dispersion corrected DFT with optB88-vdW functional.(5, 6) Geometric structures are relaxed until the force on each atom is less than 0.05 eV/Å and the convergence criteria for energy is less than 1×10^-4^ eV. All *p*(4×4) Ir(111), α-MoC(111) and Ir_1_/α-MoC(111) surfaces were modeled by a four metal layers thick slabs. Periodic slab images in the z-direction were separated by a vacuum layer of 15 Å to avoid spurious interactions between them. Adsorbates and the topmost two metal layers (including corresponding C-layers for α-MoC and Ir_1_/α-MoC) were allowed to relax. A 3 × 3 × 1 Monkhorst–Pack k-point grid for sampling the Brillouin zone was considered for adsorption energies and activation barriers calculation on the three surfaces. To search for transition states, the improved force reversed method with a force tolerance of 0.05 eV/Å was used.(7) Some transition states were verified using the climbing-image nudged elastic band method.(8, 9) We assume the intermediates can rotate freely for quinoline hydrogenation on Ir/α-MoC structure by using the single Ir atom. The adsorption energies were calculated with respect to the corresponding molecules in the gas phase. The activation barrier was calculated as the energy difference between the adsorption state and transition state. The zero-point energy correction for adsorbates was not considered. The calculated lattice constants for cubic Ir and α-MoC are 3.878 Å and 4.369 Å, respectively, which are consistent with our experimental measurements.

The formation energy (*E*_f_) of a Ir atom embedded in the surface Mo defect site in α-MoC (111) can be calculated as:

*E*_f_ = *E*(Ir/α-MoC) – *E*(α-MoC) – *E*(Ir) (S3)

where *E*(Ir/α-MoC), *E*(α-MoC) and *E*(Ir) are the total energies of Ir/α-MoC (111) surface, α-MoC (111) surface with a Mo vacancy site and a Ir atom in the bulk phase, respectively.

***Microkinetic simulation.*** Using the computed energetic as input, microkinetic simulations have been conducted to predict quinoline hydrogenation reaction rate, surface coverage distribution and degree of rate control (DRC) by means of MKMCXX software.(10-12) The approach for microkinetic simulation has been described in detail elsewhere.(10, 11) In general, the computed activation barriers were used to compute the forward and backward reaction rate constants. For the surface reactions, the rate constants for the forward and backward elementary reaction were determined by the Eyring equation(13)

$k=Ae^{-\frac{Ea}{k_{b}T}}$ (S4)

where *k* is the reaction rate constant in s^-1^. *k*_b_, *T* and *E*_a_ are the Boltzmann constant, temperature and the activation barrier, respectively. We used 10^13^ s^-1^ for the prefactor *A* as an approximation here.

For non-activated molecular adsorption, the rate of adsorption is determined by the rate of surface impingement of gas-phase molecules. Based on the Hertz-Knudsen equation,(14) we obtain the molecular adsorption rate constant as:

$k_{ads}=\frac{PA^{'}}{\sqrt{2\pi mk_{b}T}}S$ (S5)

with *P* the partial pressure of the adsorbate in the gas phase, *A’* the surface area of the adsorption site, *m* the mass of the adsorbate and *S* the sticking coefficient used 1 here.

For the desorption process, it is assumed that there are three rotational degrees of freedom and two translational degrees of freedom in the transition state. Accordingly, the rate of desorption is given by

$k_{des}=\frac{k_{b}T^{3}}{h^{3}}\frac{A^{'}(2\pi k_{b})}{\sigma\theta_{rot}}e^{-\frac{E_{des}}{k_{b}T}}$ (S6)

where *σ* and *θ* are the symmetry number and the characteristic temperature for rotation, respectively.

Differential equations for all the surface reaction intermediates were constructed using the rate constants and the set of elementary reaction steps. For each of the *M* components in the kinetic network, a single differential equation in the form

$r_{i}=\sum_{j=1}^{N} \left(k_{j}\nu_{i}^{j}\prod_{k=1}^{M} c_{k}^{\nu_{k}^{j}}\right)$ (S7)

is obtained. In this equation, *k*_j_ is the elementary reaction rate constant (see equation 3), $\nu_{i}^{j}$is the stoichiometric coefficient of component *i* in elementary reaction step *k* and *c*_k_ is the concentration of component *k* on the catalytic surface. Steady-state coverages were calculated by integrating the ordinary differential equations in time until the changes in the surface coverages were smaller than 10^-12^.

The elementary reaction steps that contribute to the rate control over the overall reaction can be determined by DRC concept introduced by Campbell et al.(15-17) For elementary step *i*, the degree of rate control X_RC_,*_i_* can be defined as

$X_{RC,i}=\frac{k_{i}}{r}\left(\frac{\partial r}{\partial k_{i}}\right)_{k_{j\neq i},K_{i}}=\left(\frac{\partial\ln r}{\partial\ln k_{i}}\right)_{k_{j\neq i},K_{i}}$ (S8)

where *k*_i_, *K*_i_ and *r* are the rate constants, the equilibrium constant for step *i* and the reaction rate, respectively.

Quinoline hydrogenation rate is predicted by microkinetic simulations using the dual-sites model for Ir_1_/α-MoC(111) structure. During microkinetic simulations, we adopted the mole ratio of quinoline, H_2_ and H_2_O reactants by 1:20:100 which is similar to our reaction conditions. The quinoline reactant and py-THQ product prefer to bind six Ir and/or Mo atoms, we thus used the normalized adsorption/desorption energies of quinoline and py-THQ per site in the microkinetic simulations.

**Figures and Tables:**

**
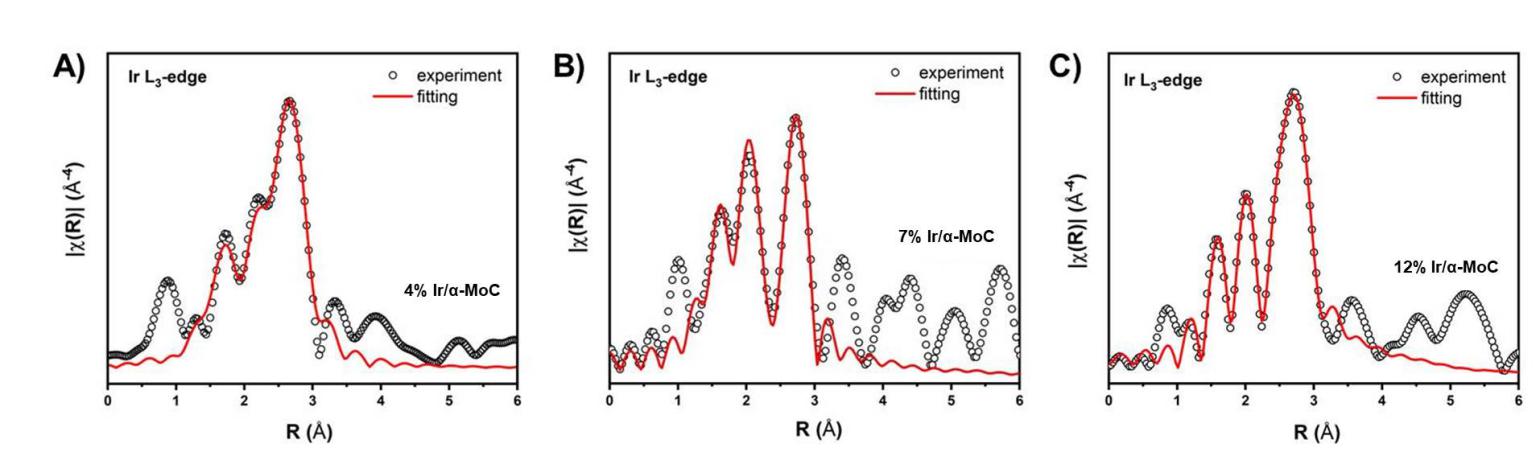
**

**Figure S1.** FT-EXAFS fitting curves of the Ir/α-MoC catalysts at Ir L_3_-edge.


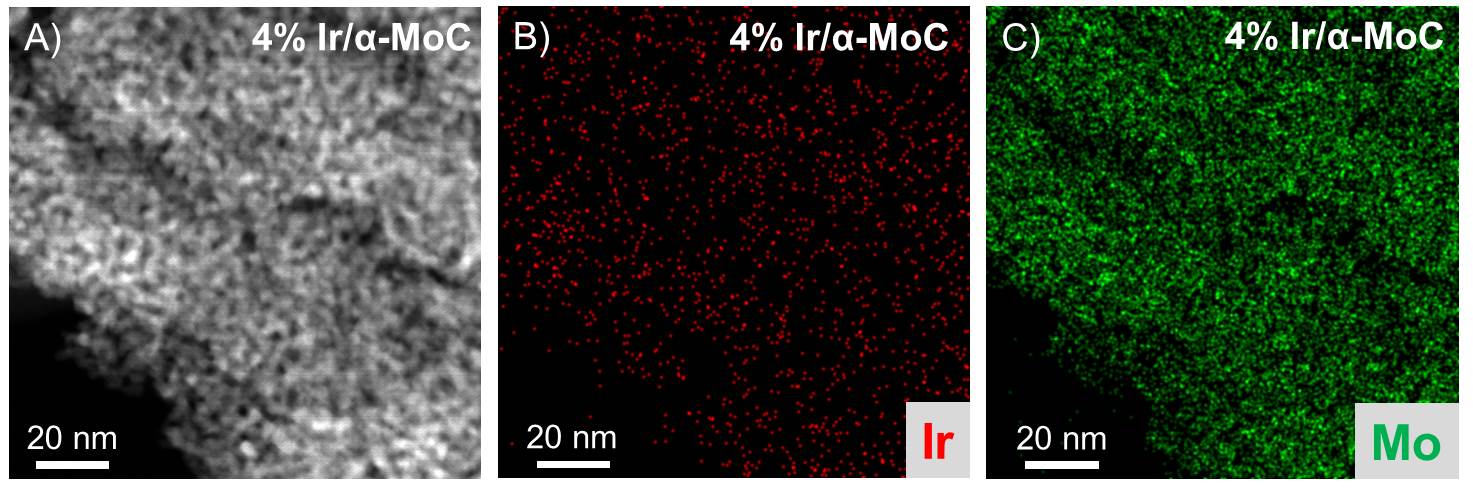


**Figure S2.** Elemental mapping of the 4% Ir/α-MoC sample. A) The STEM-HAADF image. B) The corresponding Ir Lα (9.17 keV) EDS mapping. C) The corresponding Mo Kα (17.48 keV) mapping.


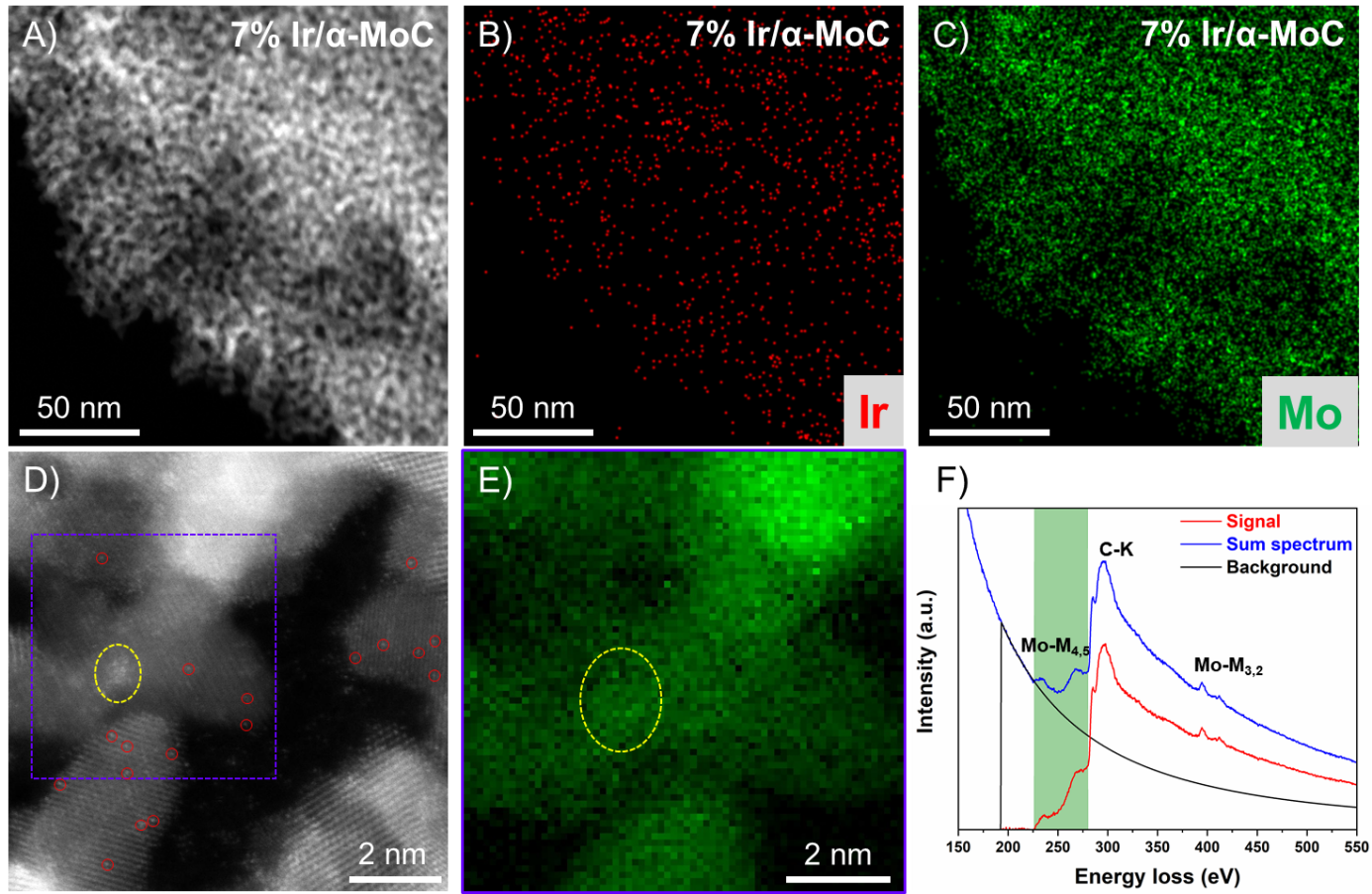


**Figure S3.** Elemental mapping of the 7% Ir/α-MoC sample. The STEM-HAADF image (A) and corresponding Ir Lα (9.17 keV) EDS mapping (B), Mo Kα (17.48 keV) mapping (C). (D) The STEM-HAADF image. (E) The elemental maps of Mo M_4,5_ EELS signal (labelled in green in (F)) of the purple rectangle in (D). (F) EELS spectrum extracted from the purple rectangle in (D). The EELS mapping in Figure S3E presents that the brighter contrast in HAADF imaging (labelled by the dashed yellow ellipse in Figure S3D) is caused by the Ir clusters, not the thickness change of α-MoC supports.


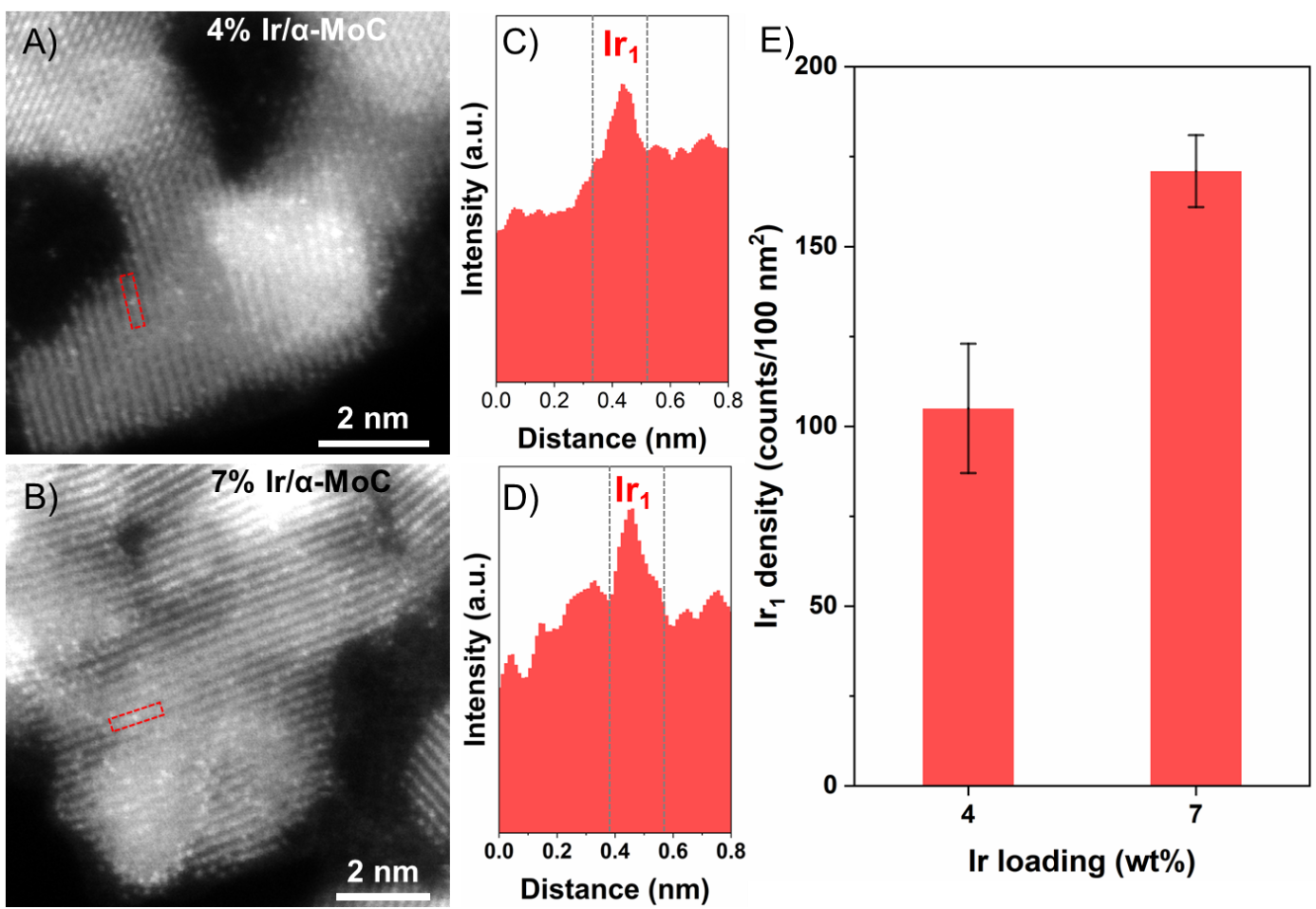


**Figure S4.** Statistical analysis of the Ir_1_ species in the 4% and 7% Ir/α-MoC samples. (A-B) High-resolution STEM-HAADF images of 4% and 7% Ir/α-MoC samples. (C-D) The corresponding intensity line profiles confirming the presence of Ir_1_ species. (E) Statistics of the density of Ir_1_ species in the 4% and 7% Ir/α-MoC samples.


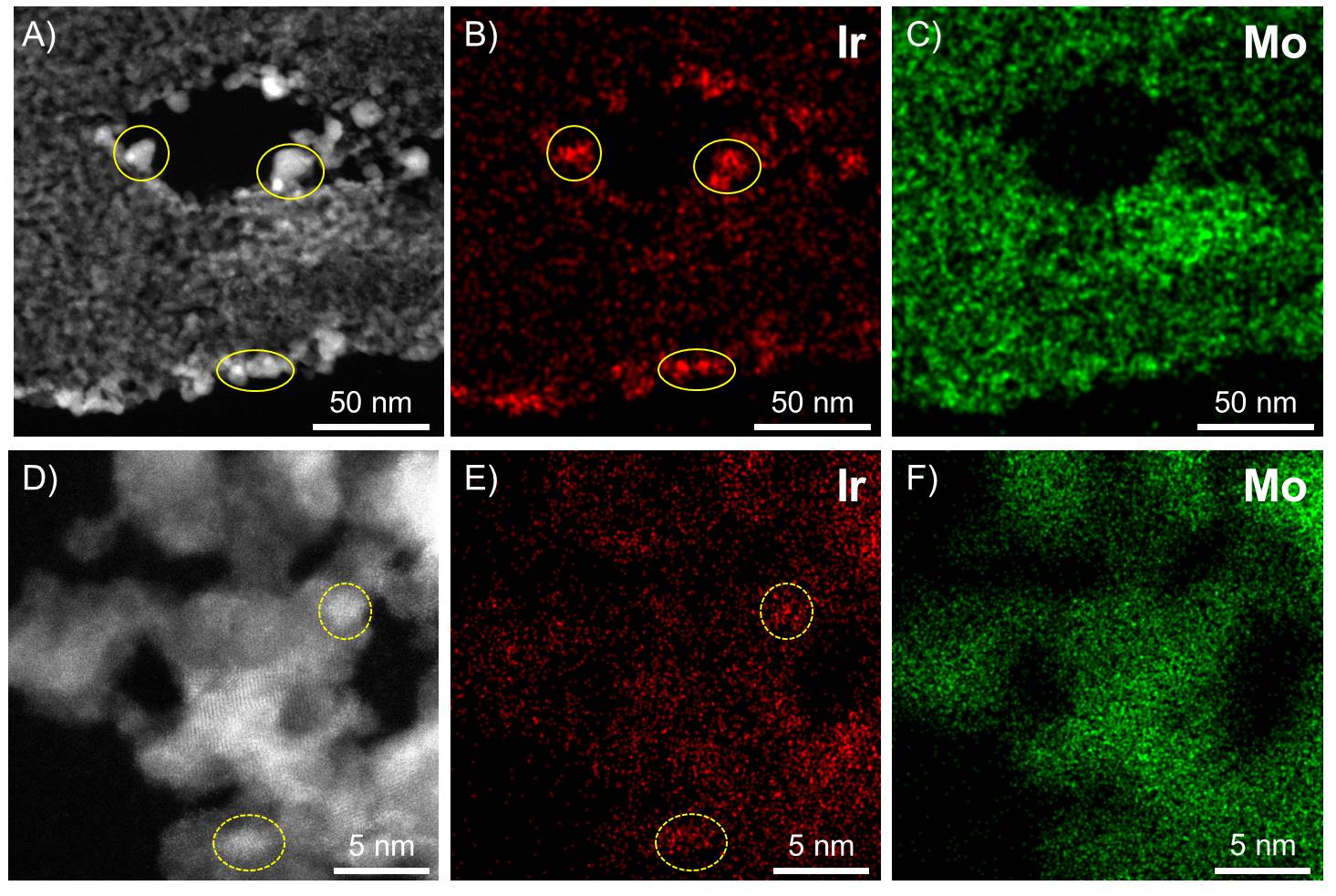


**Figure S5.** Elemental mapping of the 12% Ir/α-MoC sample. The STEM-HAADF images (A and D). The corresponding Ir Lα (9.17 keV) EDS mapping (B and E). The corresponding Mo Kα (17.48 keV) mapping (C and F). Some Ir particles (labelled in yellow circles) and small Ir clusters (labelled in dashed yellow circles) were both observed.


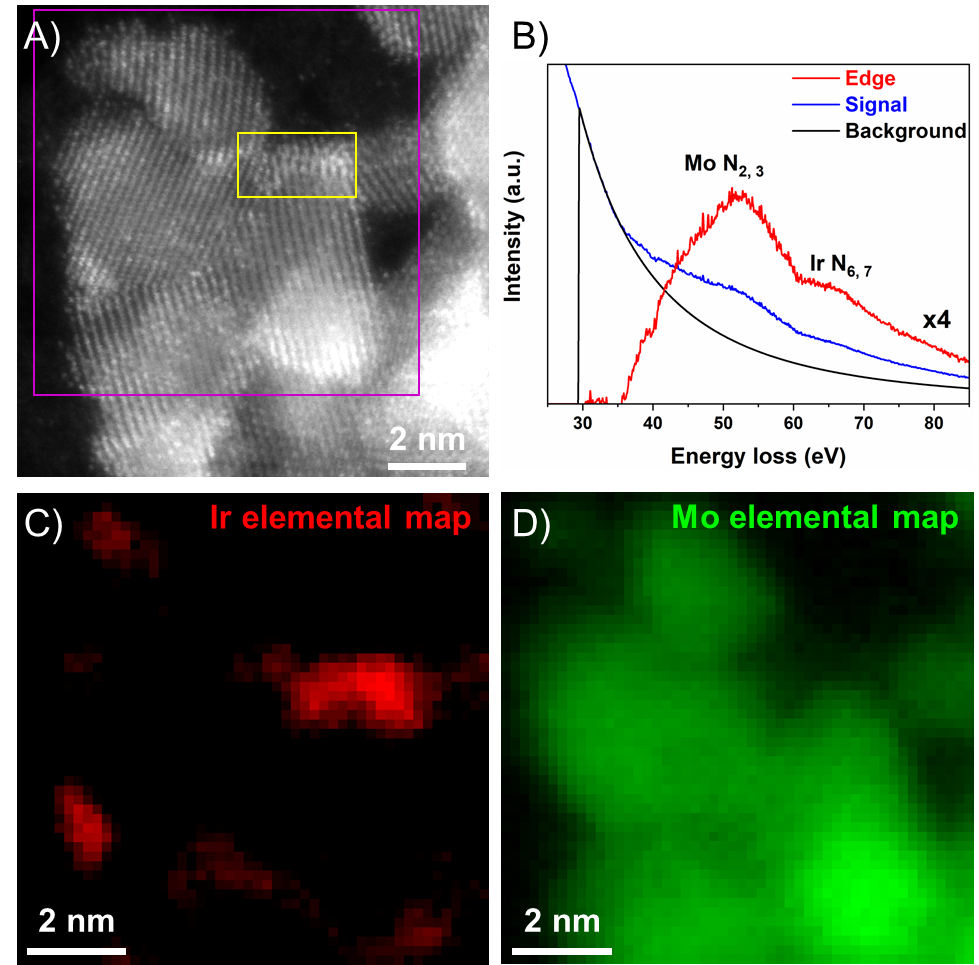


**Figure S6.** The STEM-EELS analysis of the 12% Ir/α-MoC sample. (A) STEM-HAADF image. (B) The EELS signal extracted from the yellow rectangle in (A). (C-D) The visualization of Ir and Mo elemental distribution by MLLS (multiple linear least squares) fitting of the EEL spectrum imaging acquired from the purple rectangle in (A). The EELS elemental maps provide the direct evidence for the presence of Ir clusters in the 12% Ir/α-MoC sample.


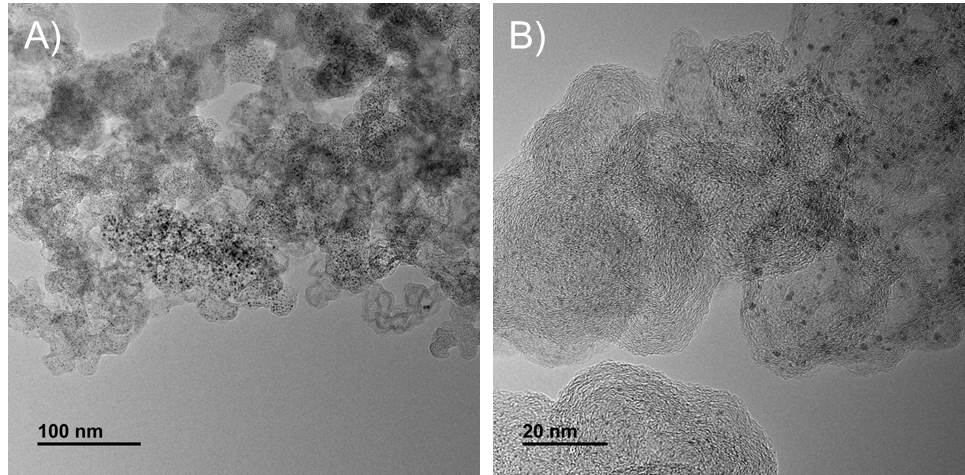


**Figure S7.** TEM images of 7% Ir/C catalyst.


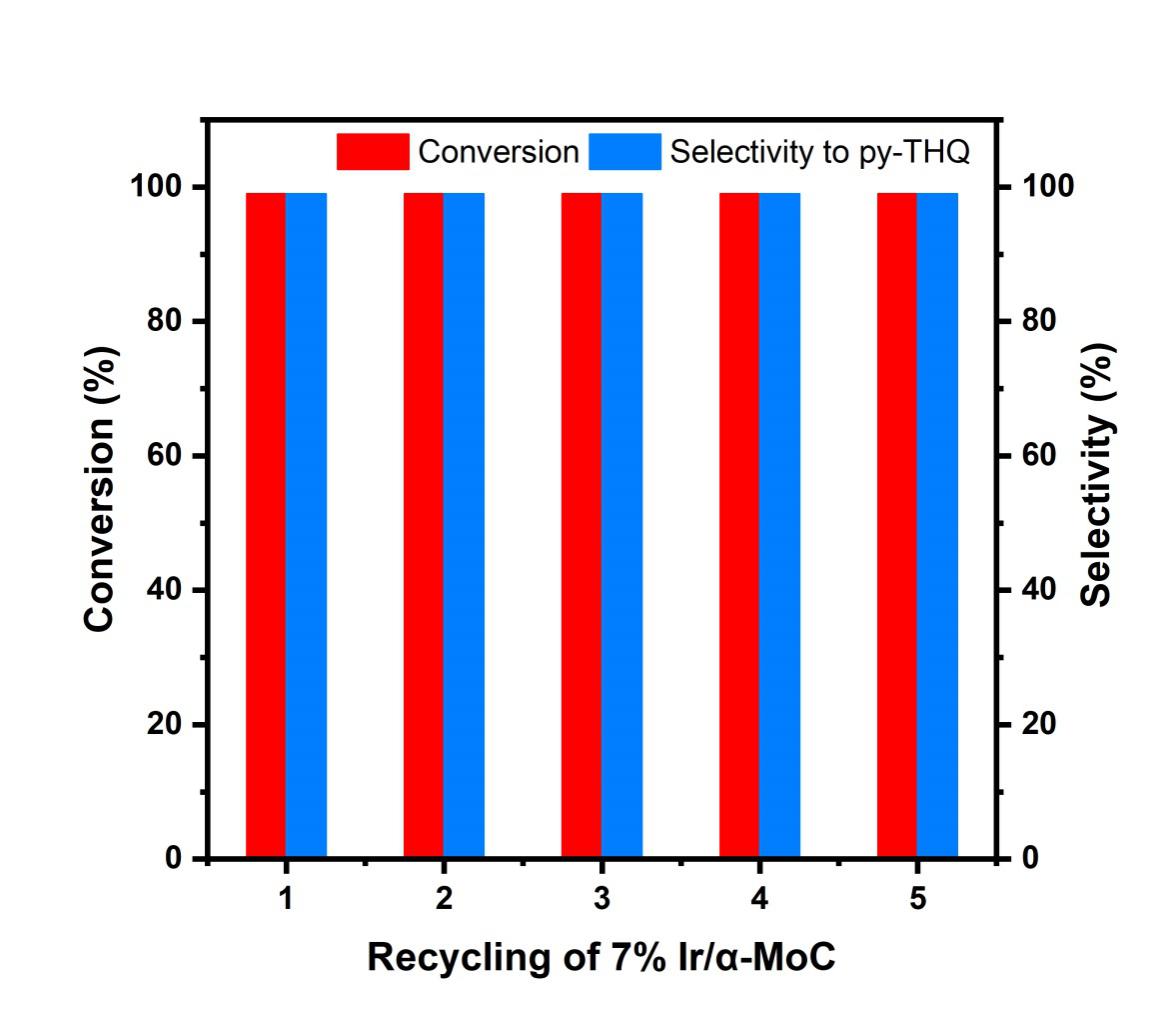


**Figure S8.** Stability test of the 7% Ir/α-MoC catalyst for quinoline hydrogenation. In the five-cycle stability test, the conversion of quinoline remained 100% with more than 99% selectivity to py-THQ. The products were analyzed by GC-MS. Reaction condition: 30 mg catalyst, 40 mg quinoline in 3 mL of CH_3_OH/H_2_O (v:v=1:1), 3 Mpa H_2_, 120 °C reaction for each 2 hrs cycle.


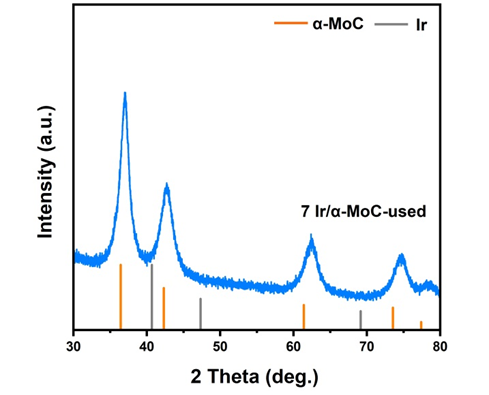


**Figure S9.** XRD of 7% Ir/α-MoC obtained after quinoline hydrogenation reaction.


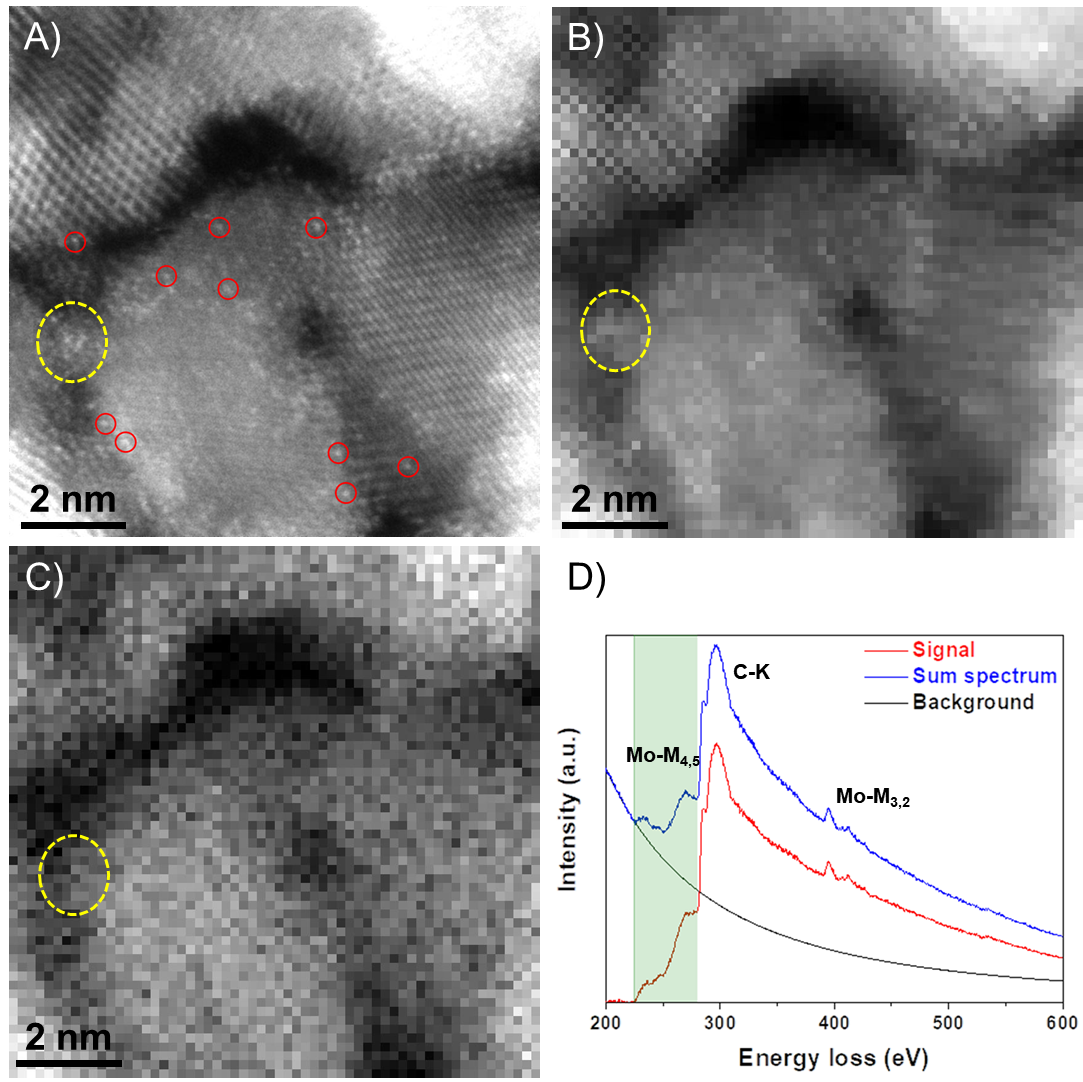


**Figure S10.** STEM analysis for the used 7% Ir/α-MoC sample. (A) Atomic resolution STEM-HAADF image. (B) HAADF image acquired simultaneously with EELS mapping. (C) The Mo-M_4,5_ EELS mapping. (D) The EELS spectrum in (B). Even after the catalytic reaction, the highly-dispersed nature of Ir species in the 7% Ir/α-MoC catalyst preserves and the Ir cluster (labelled in the dashed yellow circle) can be distinguished by EELS in Figure S10B and C.


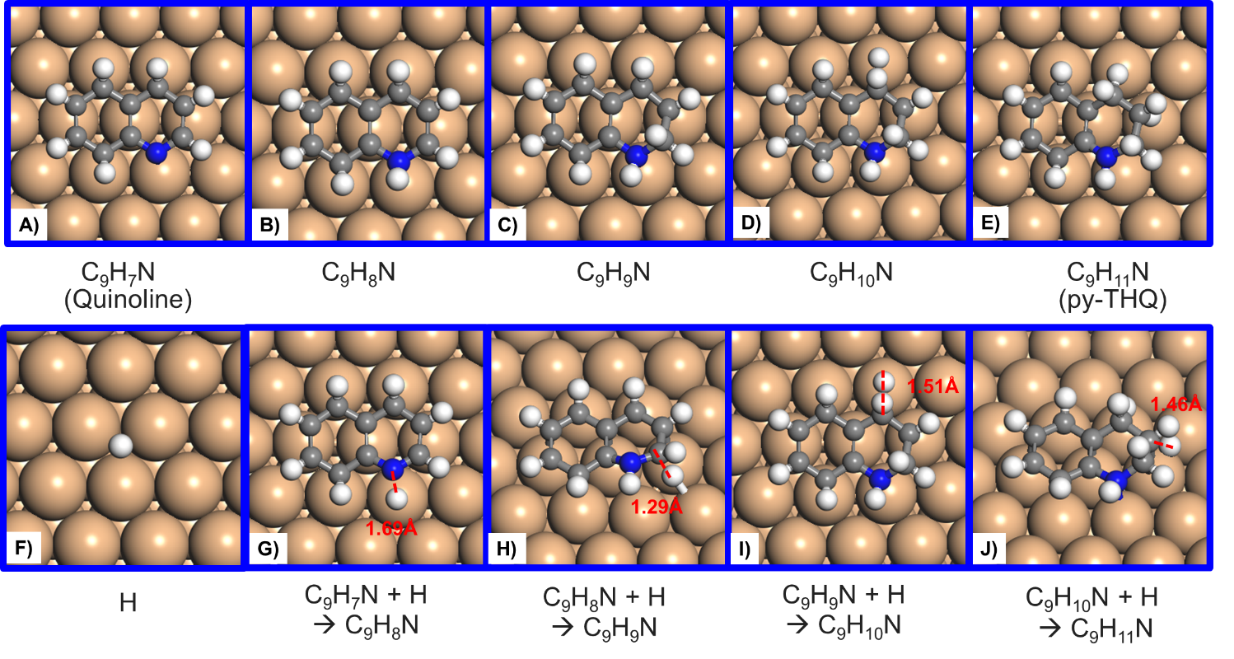


**Figure S11-1.** Adsorption (A-F) and transition states configurations (G-J) for direct quinoline hydrogenation over Ir(111) surface. The distance between hydrogen and carbon/nitrogen atoms in transition state is indicated in Å. The beige, grey, blue and white spheres are Ir, C, N and H atoms, respectively. This notation is used throughout this supporting information.


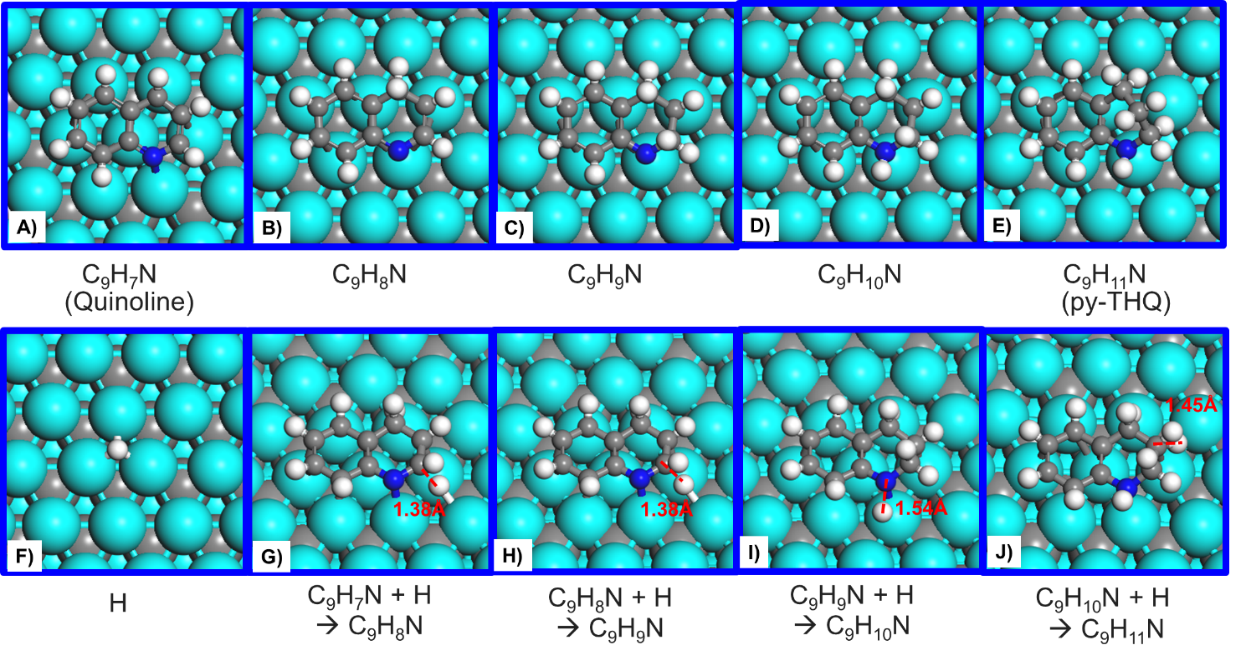


**Figure S11-2.** Adsorption (A-F) and transition states configurations (G-J) for direct quinoline hydrogenation over α-MoC(111) surface. The distance between hydrogen and carbon/nitrogen atoms in transition state is indicated in Å. The cyan spheres are Mo atoms. This notation is used throughout this supporting information.


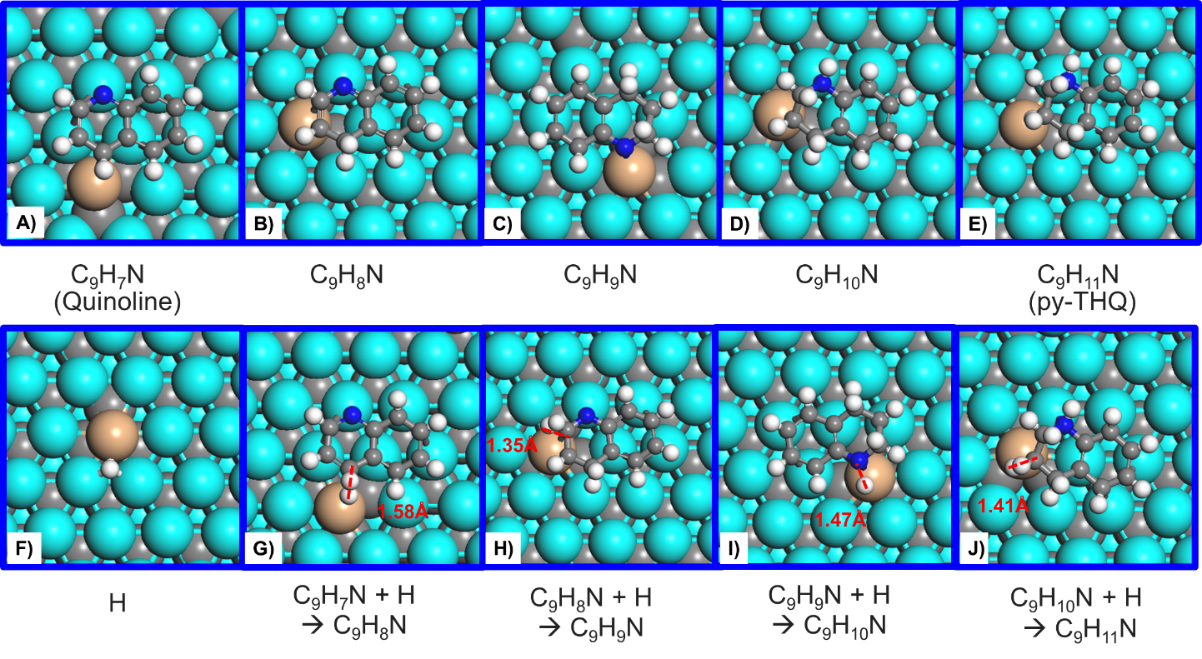


**Figure S11-3.** Adsorption (A-F) and transition states configurations (G-J) for direct quinoline hydrogenation over Ir_1_/α-MoC(111) surface The distance between hydrogen and carbon/nitrogen atoms in transition state is indicated in Å.


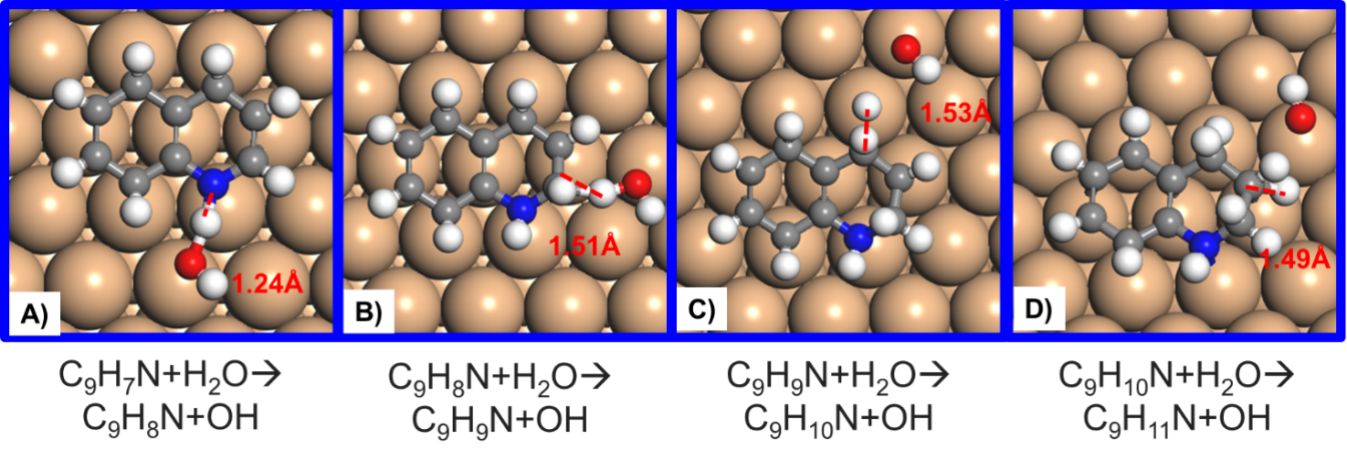


**Figure S12-1.** Optimized transition states configurations for water-mediated quinoline hydrogenation over Ir(111) surface. The distance between hydrogen and carbon/nitrogen atoms in transition state is indicated in Å.


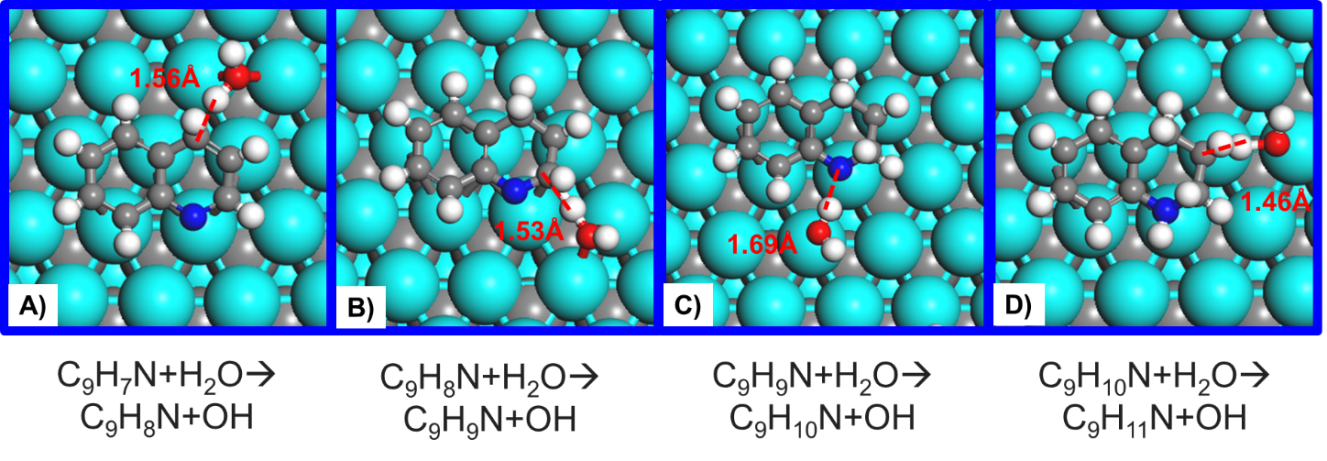


**Figure S12-2.** Optimized transition states configurations for water-mediated quinoline hydrogenation over α-MoC(111) surface. The distance between hydrogen and carbon/nitrogen atoms in transition state is indicated in Å.


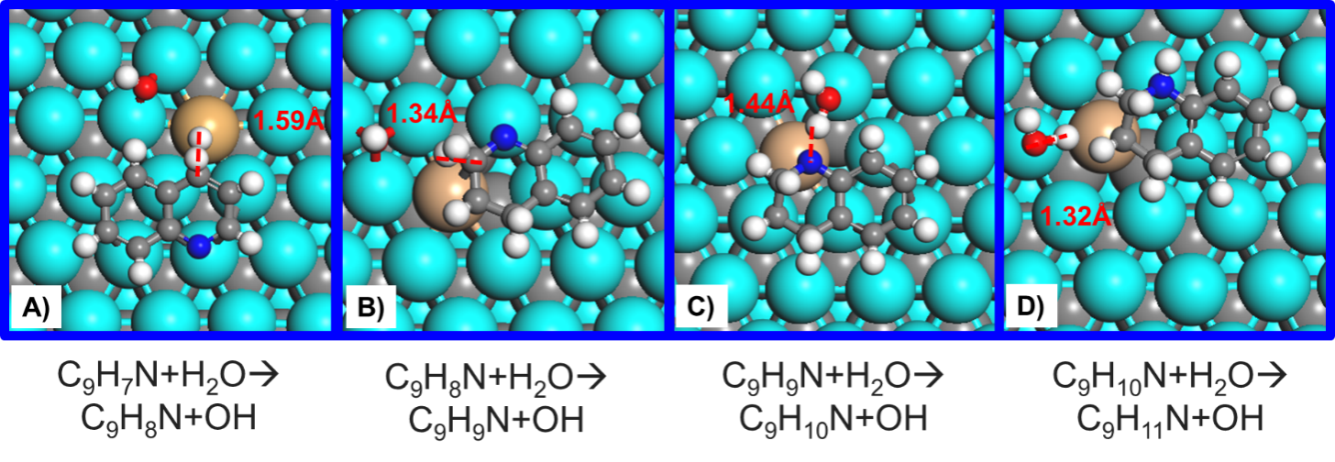


**Figure S12-3.** Optimized transition states configurations for water-mediated quinoline hydrogenation over Ir_1_/α-MoC(111) surface. The distance between hydrogen and carbon/nitrogen atoms in transition state is indicated in Å.


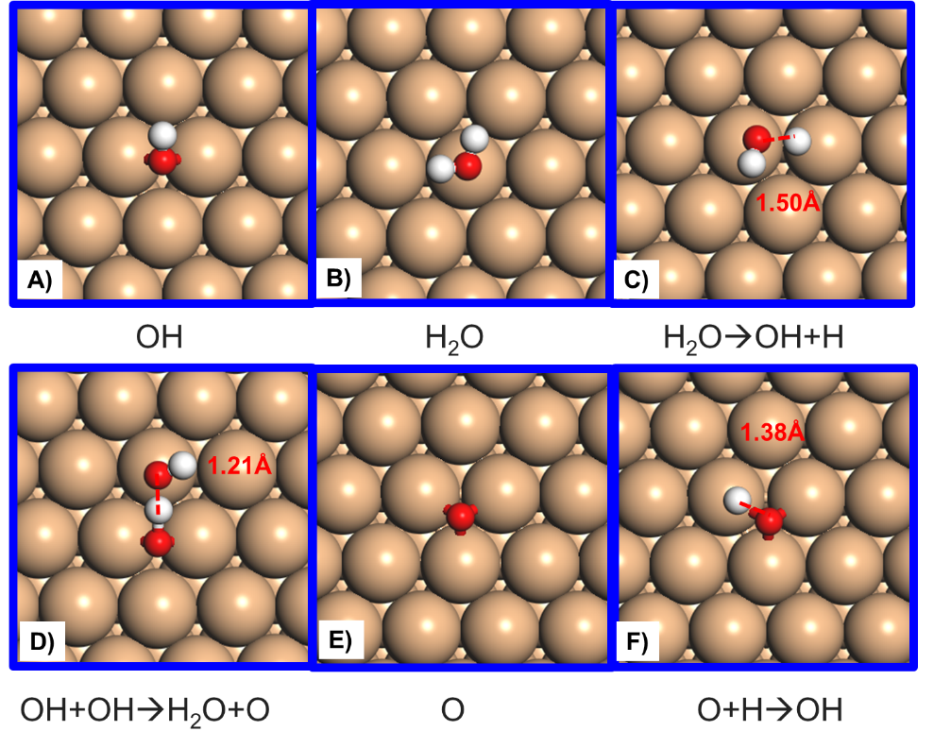


**Figure S13-1.** Calculated adsorption and transition states configurations for water formation over Ir (111) surface. The distance between hydrogen and oxygen atoms in transition state is indicated in Å.


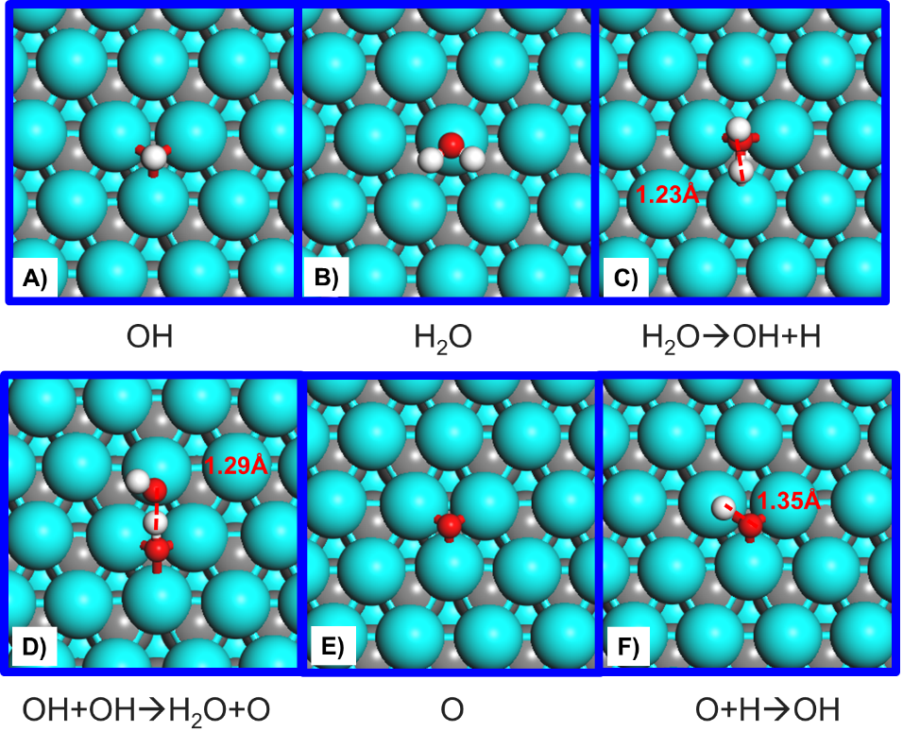


**Figure S13-2.** Calculated adsorption and transition states configurations for water formation over α-MoC(111) surface. The distance between hydrogen and carbon/nitrogen atoms in transition state is indicated in Å.


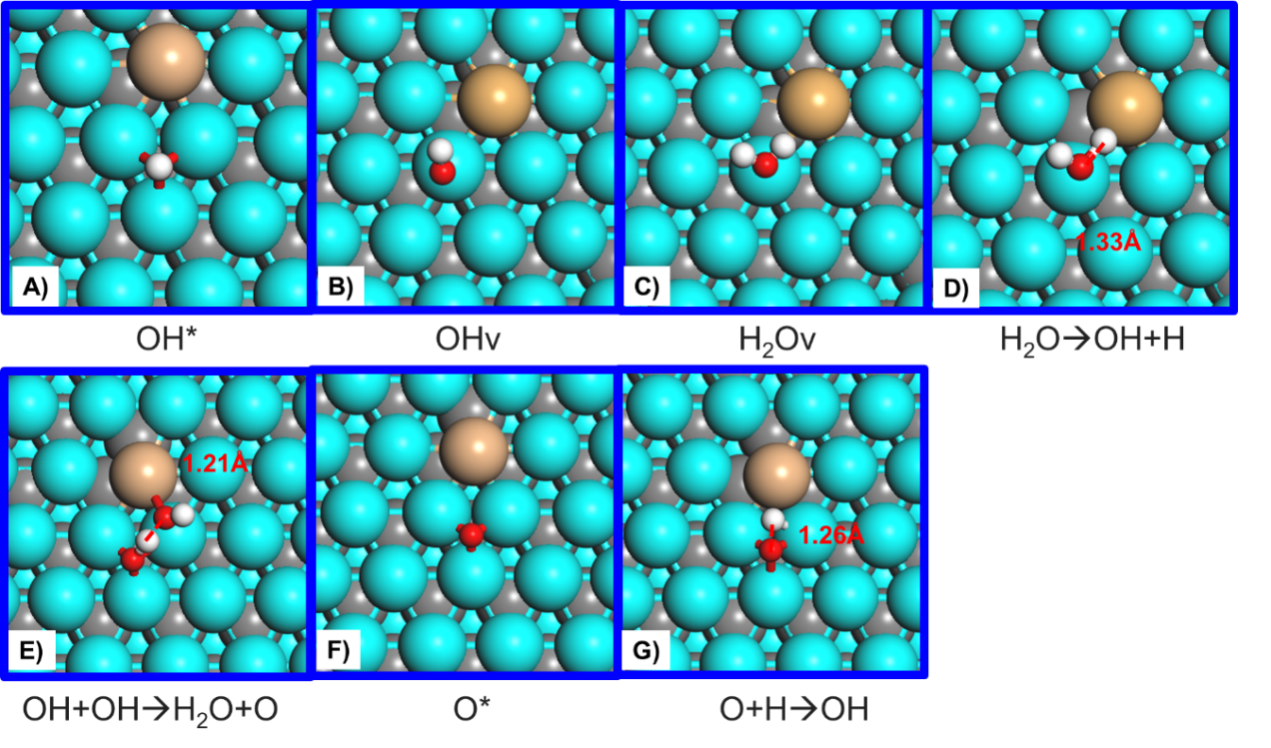


**Figure S13-3.** Calculated adsorption and transition states configurations for water formation over Ir_1_/α-MoC(111) surface. The distance between hydrogen and carbon/nitrogen atoms in transition state is indicated in Å.

**Figure S14.** Surface coverage and degree of rate control analysis for quinoline hydrogenation over Ir (111), α-MoC (111) and Ir_1_/α-MoC(111) surfaces by microkinetic simulations. Qx (x = 0 – 4) indicate C_9_H_7+x_N species. * and v are the active sites at α-MoC and Ir, respectively. # stands for Qx (x = 0 – 4) adsorption by binding six Ir or Mo atoms.

**
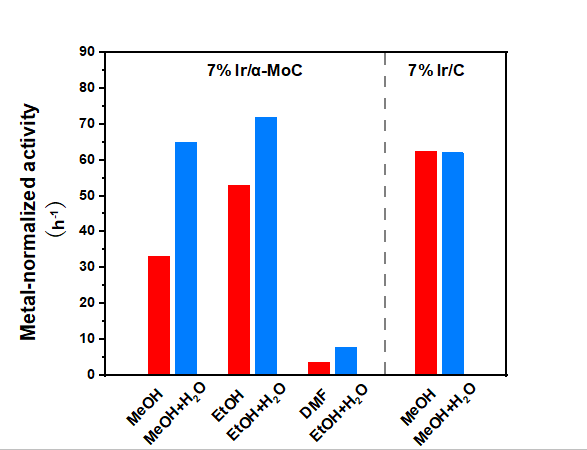
**

**Figure S15.** Comparison of the metal-normalized activity for quinoline hydrogenation reaction using neat and watered solvents. The results show that water promotes the hydrogenation of quinoline over Ir/α-MoC catalyst, but does not work for the Ir/C catalyst.


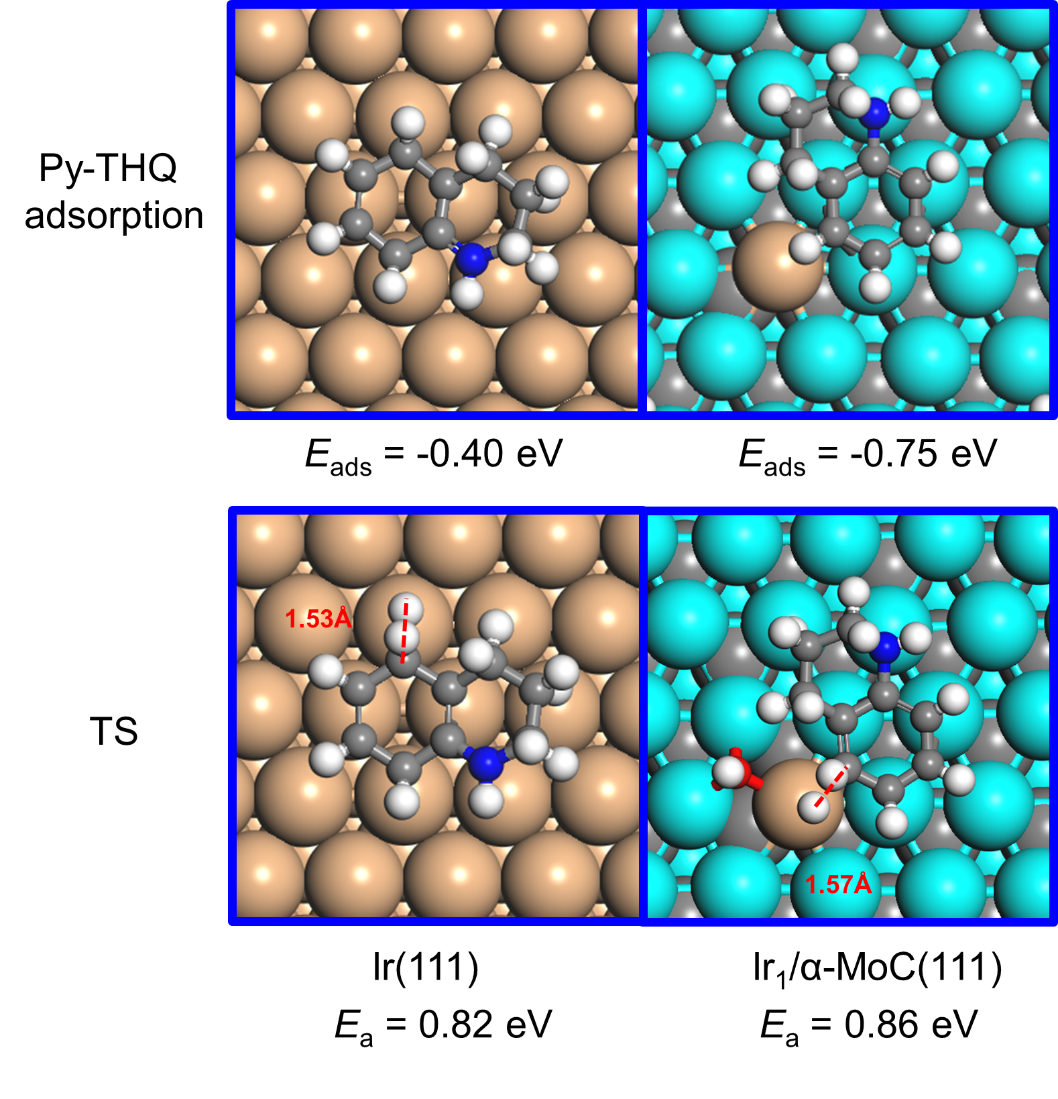


**Figure S16.** py-THQ adsorption and transition states configurations for py-THQ hydrogenation over Ir(111) and Ir_1_/α-MoC(111) surfaces. The distance between hydrogen and carbon atoms in transition state is indicated in Å. The normalized py-THQ adsorption energy (*E*_ads_) per site and py-THQ hydrogenation activation barrier (*E*_a_) are given in eV unit.

**Table S1.** EXAFS data fitting results of Ir/α-MoC catalysts at the Ir L_3_-edge.

| Sample | Shell | Bond length | C.N. | σ^2^ | E_0_ shift |
| --- | --- | --- | --- | --- | --- |
| 4% Ir/α-MoC | Ir-C | 2.05 (3) | 1.4±0.7 | 0.011 | 9.1 |
|  | Ir-Mo | 2.82 (1) | 7.3±1.1 | 0.014 |  |
| 7% Ir/α-MoC | Ir-C | 2.04 (1) | 3.5±0.4 | 0.013 | 5.1 |
|  | Ir-Ir | 2.70 (1) | 2.6±0.8 | 0.004 |  |
|  | Ir-Mo | 2.72 (1) | 3.4±0.8 | 0.008 |  |
| 12% Ir/α-MoC | Ir-C | 2.07 (2) | 3.0±1.0 | 0.009 | 9.6 |
|  | Ir-Ir | 2.72 (1) | 4.2±0.8 | 0.003 |  |
|  | Ir-Mo | 2.74 (2) | 1.0±0.2 | 0.003 |  |

**Table S2.** Calculation of metal-normalized activity (MNA) and mass-specific activity (MSA) of Ir/α-MoC and 7% Ir/C catalysts.^a^

| Entry | Catalyst | Ir loading (%)^b^ | m_substrate_ (mg) | Conv. (%) | MNA (mol_Q_ mol_Ir_^-1^  h^-1^) | MSA (μmol _Q_g^-1^ s^-1^) |
| --- | --- | --- | --- | --- | --- | --- |
| 1 | 0.5% Ir/α-MoC^c^ | 0.35 | 40 | 16 | 91 | 0.46 |
| 2 | 1% Ir/α-MoC | 1.1 | 60 | 20 | 89 | 1.3 |
| 3 | 3% Ir/α-MoC | 2.2 | 150 | 17 | 86 | 2.7 |
| 4 | 4% Ir/α-MoC | 4.0 | 240 | 19 | 85 | 4.9 |
| 5 | 7% Ir/α-MoC | 6.8 | 300 | 20 | 65 | 6.5 |
| 6 | 12% Ir/α-MoC | 12.4 | 300 | 13 | 23 | 4.3 |
| 7 | 7% Ir/C | 7.6 | 300 | 21 | 62 | 6.8 |

[a] Reaction condition: catalyst (20 mg), 3.0 MPa of H_2_. 3 mL of CH_3_OH/H_2_O (v:v=1:1) as solvent, 3.0 MPa of H_2_.

[b] Ir loading were measured with ICP-AES.

[c] For 0.5% Ir/α-MoC, 30 mg of catalyst was added.

**Table S3**. Summaries of catalysts for quinoline hydrogenation to py-THQ. Metal-normalized activity (MNA) in this table is calculated at high conversion of quinoline.

| Catalyst | T (^o^C) | P  (MPa) | Conv. | MNA (  h^-1^) | Stability | Reference |
| --- | --- | --- | --- | --- | --- | --- |
| 7% Ir/α-MoC | 120 | 3 | 95 | 27 | 99% conversion, 99% selectivity for 5 cycles | *This work* |
| Ru SAs-NC | 100 | 3.5 | 99 | 28 | 99% conversion, 99% selectivity for 5 cycles | *J. Am. Chem. Soc.* 2017, 139, 9419 |
| Au/HAS-TiO_2_ | 60 | 2 | 100 | 29 | 97% yield for 2 cycles | *J. Am. Chem. Soc.* 2012, 134, 17592 |
| Pd-polymer | 80 | 1 | 98 | 22 | - | *Appl. Catal. A* 2014, 481, 89 |
| Pt/*NR*-CeO_2_ | 25 | 2 | 99 | 24 | - | *J. Catal.* 2018, 359, 101 |
| Ru/SiO_2_@m SiO_2_ | 90 | 2 | 30.5 | 30 | 99% conversion, 99% selectivity for 5 cycles.  35.2% conversion 99% selectivity after 5 cycles | *Catal. Sci. Technol.* 2014*,* 4, 1939 |
| CoO_x_@CN | 120 | 3 | 95 | 4.1 | 89% yield after 10 cycles, 57% yield after 12 cycles | *ACS Catal. 2016, 6, 5816−5822* |
| BWT-stabilized Pd | 80 | 2 | 96 | 192 | 71.1% conversion after 3 cycles | *Catal. Sci. Technol., 2013, 3, 1612--1617* |

**Table S4.** Ir/MoC catalyzed hydrogenation of quinoline derivatives and isoquinoline.^a^


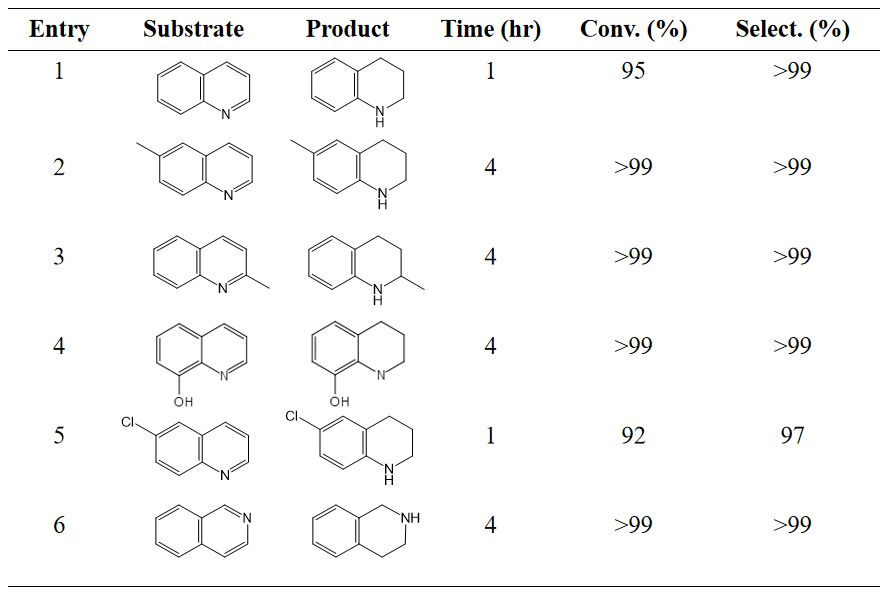


1. reaction condition: catalyst (30 mg), 40 mg substrate, 3 mL of CH_3_OH/H_2_O (v:v=1:1) as solvent, 3.0 MPa of H_2_.

**Table S5.** Calculated adsorption energies of quinoline (*E*_Q_), py-THQ (*E*_py-THQ_) and hydrogen (*E*_H_), and elementary forward (*E*_if_, i = 1 – 4) and backward (*E*_bf_, i = 1 – 4) activation barriers for direct quinoline hydrogenation towards py-THQ. All the energies are in eV unit.

| Structures | *E*_Q_ | *E*_py-THQ_ | *E*_H_ | *E*_1f_ | *E*_1b_ | *E*_2f_ | *E*_2b_ | *E*_3f_ | *E*_3b_ | *E*_4f_ | *E*_4b_ |
| --- | --- | --- | --- | --- | --- | --- | --- | --- | --- | --- | --- |
| Ir(111) | – 2.71 | –2.38 | –0.29 | 0.67 | 0.80 | 1.01 | 1.10 | 0.73 | 0.69 | 0.90 | 0.68 |
| α-MoC(111) | –5.09 | –4.19 | –0.88 | 1.49 | 1.07 | 2.24 | 1.31 | 2.18 | 1.66 | 1.76 | 0.67 |
| Ir_1_/α-MoC(111) | –5.37 | –3.71 | –0.68 | 1.18 | 0.15 | 1.62 | 0.69 | 1.52 | 1.41 | 1.40 | 0.55 |

**Table S6.** Calculated forward (*E*_if_, i = 1 – 4) and backward (*E*_ib_, i = 1 – 4) activation barriers for water-mediated quinoline hydrogenation towards py-THQ. All the energies are in eV unit.

| Structures | *E*_1f_ | *E*_1b_ | *E*_2f_ | *E*_2b_ | *E*_3f_ | *E*_3b_ | *E*_4f_ | *E*_4b_ |
| --- | --- | --- | --- | --- | --- | --- | --- | --- |
| Ir(111) | 0.47 | 0.12 | 1.88 | 1.50 | 1.14 | 0.61 | 1.28 | 0.57 |
| α-MoC(111) | 0.98 | 2.22 | 1.36 | 2.08 | 0.00 | 1.13 | 0.99 | 1.55 |
| Ir_1_/α-MoC(111) | 0.03 | 0.20 | 0.61 | 0.86 | 0.13 | 1.21 | 0.31 | 0.65 |

**Table S7.** Calculated adsorption energies of water/hydroxyl/oxygen species and forward (*E*_f_) and backward (*E*_b_) activation barriers for water formation. * and v stand for active site on α-MoC and Ir, respectively. All the energies are in eV unit.

| Structures | *E*_H2O_ | *E*_OH_ | *E*_O_ | OH* + H*  🡪 H_2_O* + * | | OH* + OH*  🡪 H_2_O* + O* | | O* + H*  🡪 OH* + * | |
| --- | --- | --- | --- | --- | --- | --- | --- | --- | --- |
|  |  |  |  | *E*_f_ | *E*_b_ | *E*_f_ | *E*_b_ | *E*_f_ | *E*_b_ |
| Ir(111) | –0.54 | –2.82 | -4.96 | 0.38 | 0.86 | 0.00 | 0.19 | 1.12 | 1.11 |
| α-MoC(111) | –0.99 | –4.81 | -7.19 | 2.19 | 0.54 | 1.03 | 0.22 | 1.98 | 1.14 |
| Structures | *E*_H2O_ | *E*_OH_ | *E*_O_ | OHv + Hv  🡪 H_2_Ov + v | | OH* + OHv  🡪 H_2_Ov + O* | | O* + Hv  🡪 OH* +v | |
|  |  |  |  | *E*_f_ | *E*_b_ | *E*_f_ | *E*_b_ | *E*_f_ | *E*_b_ |
| Ir_1_/α-MoC(111) | –1.00 | –4.57 | -7.07 | 1.00 | 0.37 | 0.54 | 0.66 | 1.65 | 0.91 |

**Table S8.** Bader charge (*Q*_Ir_ and/or *Q*_Mo_) of surface Ir and Mo atom in Ir(111), α-MoC(111) and Ir_1_/α-MoC(111) structures. The charge transfer from catalyst surface to quinoline is shown for quinoline adsorption.

| Structures | *Q*_Ir_ | *Q*_Mo_ | *Ɛ*_Mo_ (eV) | *Q*_Transfer_ |
| --- | --- | --- | --- | --- |
| Ir(111) | –0.03 | – | – | –0.59 |
| α-MoC(111) | – | +1.30 | –3.54 | –2.10 |
| Ir_1_/α-MoC(111) | –0.17 | +1.44 | –3.39 | –1.84 |

**References:**

1. Blöchl, PE. Projector augmented-wave method. *Phys Rev B*. 1994; **50**(24): 17953.

2. Perdew, JP, Burke, K, Ernzerhof, M. Generalized gradient approximation made simple. *Phys Rev Lett*. 1996; **77**(18): 3865-8.

3. Kresse, G, Hafner, J. Ab initio molecular dynamics for liquid metals. *Phys Rev B*. 1993; **47**(1): 558-61.

4. Kresse, G, Furthmüller, J. Efficient iterative schemes for ab initio total-energy calculations using a plane-wave basis set. *Phys Rev B*. 1996; **54**(16): 11169-86.

5. Klimeš, J, Bowler, DR, Michaelides, A. Chemical accuracy for the van der Waals density functional. *J Phys: Condens Matter*. 2009; **22**(2): 022201.

6. Klimeš, J, Bowler, DR, Michaelides, A. Van der Waals density functionals applied to solids. *Phys Rev B*. 2011; **83**(19): 195131.

7. Sun, K, Zhao, Y, Su, HY*, et al.* Force reversed method for locating transition states. *Theor Chem Acc*. 2012; **131**(2): 1-10.

8. Henkelman, G, Uberuaga, BP, Jónsson, H. A climbing image nudged elastic band method for finding saddle points and minimum energy paths. *J Chem Phys*. 2000; **113**: 9901.

9. Henkelman, G, Jónsson, H. Improved tangent estimate in the nudged elastic band method for finding minimum energy paths and saddle points. *J Chem Phys*. 2000; **113**: 9978.

10. Filot, IAW, van Santen, RA, Hensen, EJM. The optimally performing Fischer–Tropsch catalyst. *Angew Chem*. 2014; **126**(47): 12960-4.

11. Filot, IA, Broos, RJ, van Rijn, JP*, et al.* First-principles-based microkinetics simulations of synthesis gas conversion on a stepped rhodium surface. *ACS Catal*. 2015; **5**(9): 5453-67.

12. <http://www.mkmcxx.nl/>.

13. Eyring, H. The activated complex in chemical reactions. *J Chem Phys*. 1935; **3**(2): 107-15.

14. Nitoń, P, Żywociński, A, Fiałkowski, M*, et al.* A “nano-windmill” driven by a flux of water vapour: a comparison to the rotating ATPase. *Nanoscale*. 2013; **5**(20): 9732-8.

15. Campbell, CT. Future directions and industrial perspectives micro-and macro-kinetics: their relationship in heterogeneous catalysis. *Top Catal*. 1994; **1**(3-4): 353-66.

16. Campbell, CT. Finding the rate-determining step in a mechanism: Comparing DeDonder relations with the “degree of rate control”. *J Catal*. 2001; **204**(2): 520-4.

17. Stegelmann, C, Andreasen, A, Campbell, CT. Degree of rate control: How much the energies of intermediates and transition states control rates. *J Am Chem Soc*. 2009; **131**(23): 8077-82.
